# A platform for discovery of functional cell-penetrating peptides for efficient multi-cargo intracellular delivery

**DOI:** 10.1038/s41598-018-30790-2

**Published:** 2018-08-22

**Authors:** Katrin Hoffmann, Nadia Milech, Suzy M. Juraja, Paula T. Cunningham, Shane R. Stone, Richard W. Francis, Mark Anastasas, Clinton M. Hall, Tatjana Heinrich, Heique M. Bogdawa, Scott Winslow, Marie N. Scobie, Robert E. Dewhurst, Laura Florez, Ferrer Ong, Maria Kerfoot, Danie Champain, Abbie M. Adams, Susan Fletcher, Helena M. Viola, Livia C. Hool, Theresa Connor, Brooke A. C. Longville, Yew-Foon Tan, Karen Kroeger, Volker Morath, Gregory A. Weiss, Arne Skerra, Richard M. Hopkins, Paul M. Watt

**Affiliations:** 1 Telethon Kids Institute, University of Western Australia, Subiaco, Western Australia Australia; 2 Phylogica Pty Ltd, Subiaco, Western Australia Australia; 3 Centre for Comparative Genomics, Murdoch University, Perth, WA 6150 Australia; 4 School of Human Sciences, The University of Western Australia, Crawley, WA Australia; 5 Lehrstuhl für Biologische Chemie, Technische Universität München, 85354 Freising-Weihenstephan, Germany; 6 Victor Chang Cardiac Research Institute, Sydney, NSW Australia; 7 Perron Institute for Neurological and Translational Science and Centre for Neuromuscular and Neurological Disorders, The University of Western Australia, Nedlands, WA 6009 Australia; 8 University of California, Irvine, Department of Molecular Biology and Biochemistry, Irvine, CA 92697-2025 USA

## Abstract

Cell penetrating peptides (CPPs) offer great potential to deliver therapeutic molecules to previously inaccessible intracellular targets. However, many CPPs are inefficient and often leave their attached cargo stranded in the cell’s endosome. We report a versatile platform for the isolation of peptides delivering a wide range of cargos into the cytoplasm of cells. We used this screening platform to identify multiple “Phylomer” CPPs, derived from bacterial and viral genomes. These peptides are amenable to conventional sequence optimization and engineering approaches for cell targeting and half-life extension. We demonstrate potent, functional delivery of protein, peptide, and nucleic acid analog cargos into cells using Phylomer CPPs. We validate *in vivo* activity in the cytoplasm, through successful transport of an oligonucleotide therapeutic fused to a Phylomer CPP in a disease model for Duchenne’s muscular dystrophy. This report thus establishes a discovery platform for identifying novel, functional CPPs to expand the delivery landscape of druggable intracellular targets for biological therapeutics.

## Introduction

Cell penetrating peptides (CPPs) can transport therapeutic cargos directly into cells. Traditionally, CPPs are defined as relatively short (10–30 amino acids, aa), water-soluble, cationic or amphipathic peptides that can deliver a wide variety of molecules across cellular membranes^1,2^. These cargos have included biologics such as proteins, oligonucleotides, nanoparticles and small molecule drugs^3,4^. CPPs are broadly categorized into three main groups according to their origin: protein-derived, chimeric, and synthetic. Other characteristics can be used to sub-classify CPPs, usually based on their specific origin (e.g., antimicrobial) or biophysical characteristics (e.g., amphipathic)^5^.

Despite identification of over one thousand unique CPPs to date^6,7^, few CPP-linked drugs have entered the clinic^8,9^. Most clinical trials have involved TAT, a CPP derived from the HIV transactivator protein^8,10^. However, numerous pre-clinical studies have reported delivery of fluorophore-labeled CPPs or CPP-cargo fusions into cells using fluorescence microscopy^11–14^. Closer analysis reveals that these CPPs are not generally efficient at delivering cargo into the cytoplasm; instead, the CPP-cargo fusions remain largely trapped within endosomes^11,15–17^. This constitutes a key bottleneck greatly limiting cytoplasmic delivery and the resultant feasibility for therapeutic applications. Experiments estimating protein uptake suggest that at least 90% of TAT-fused cargo remains trapped within the endosomes, and is not released to the cytoplasm^11,15,18^. Despite this, at high concentrations (≥20 µM), cationic CPPs can show high intracellular uptake levels caused by non-specific flooding via non-endocytotic pathways^19^. However only limited clinical applications exist for CPPs that require such high concentrations to trigger the dose-threshold of the uptake process.

Traditional solutions to improve CPP potency and reduce dosing thresholds have relied on two strategies. First, amino acid modifications can be introduced into the CPP sequence^20^. Second, endosomolytic agents can be included either in *trans* or in *cis* with respect to the CPP-cargo fusion; for example, fusion with the HA2 sequence from influenza can improve cellular uptake^11,21^. More recently, alternative approaches to improve uptake potency have included dimerization of TAT^22^, cyclization^23^, the addition of cell binding peptides^24^, and the use of synthetic endosomal escape domains^25^ or adaptors^26^. These approaches can improve delivery into the cytoplasm to varying degrees. However, a key challenge for CPP research remains the identification of new CPPs with greater innate delivery performance. Furthermore, new CPPs must also be compatible with standard optimization approaches to enhance drug-like properties of biologics, such as the addition of moieties to increase half-life or confer tissue targeting.

Here, we address this challenge using Phylomer peptide libraries^27,28^. These small protein fragments are derived from biodiverse genomes, a potentially rich source of stable and therapeutically relevant peptides. We have successfully screened these libraries against intracellular protein targets as well as directly in phenotypic screens^29–31^. Since pathogenic bacteria and viruses have evolved sequences to facilitate transport through cell membranes^32^, we hypothesized that adding fragments from the genomes of such species into Phylomer libraries could provide novel CPPs. This expectation motivated the development and application of a new CPP discovery platform that selects and evolves CPPs based on successful, functional delivery into the cytoplasm of cells. We show that screens of Phylomer libraries yield multiple CPPs and functional validation demonstrates Phylomer CPPs are able to successfully deliver a wide range of different cargo classes into the cytoplasm of various cell types. The efficiency of Phylomer CPPs to deliver biologics offers a new path to improved therapeutic potency and reduced dosing thresholds. Thus, both the approach and the peptides reported here have great potential to expand the intracellular landscape of druggable targets.

## Results

### A novel screening platform for CPP discovery

**Figure 1:**
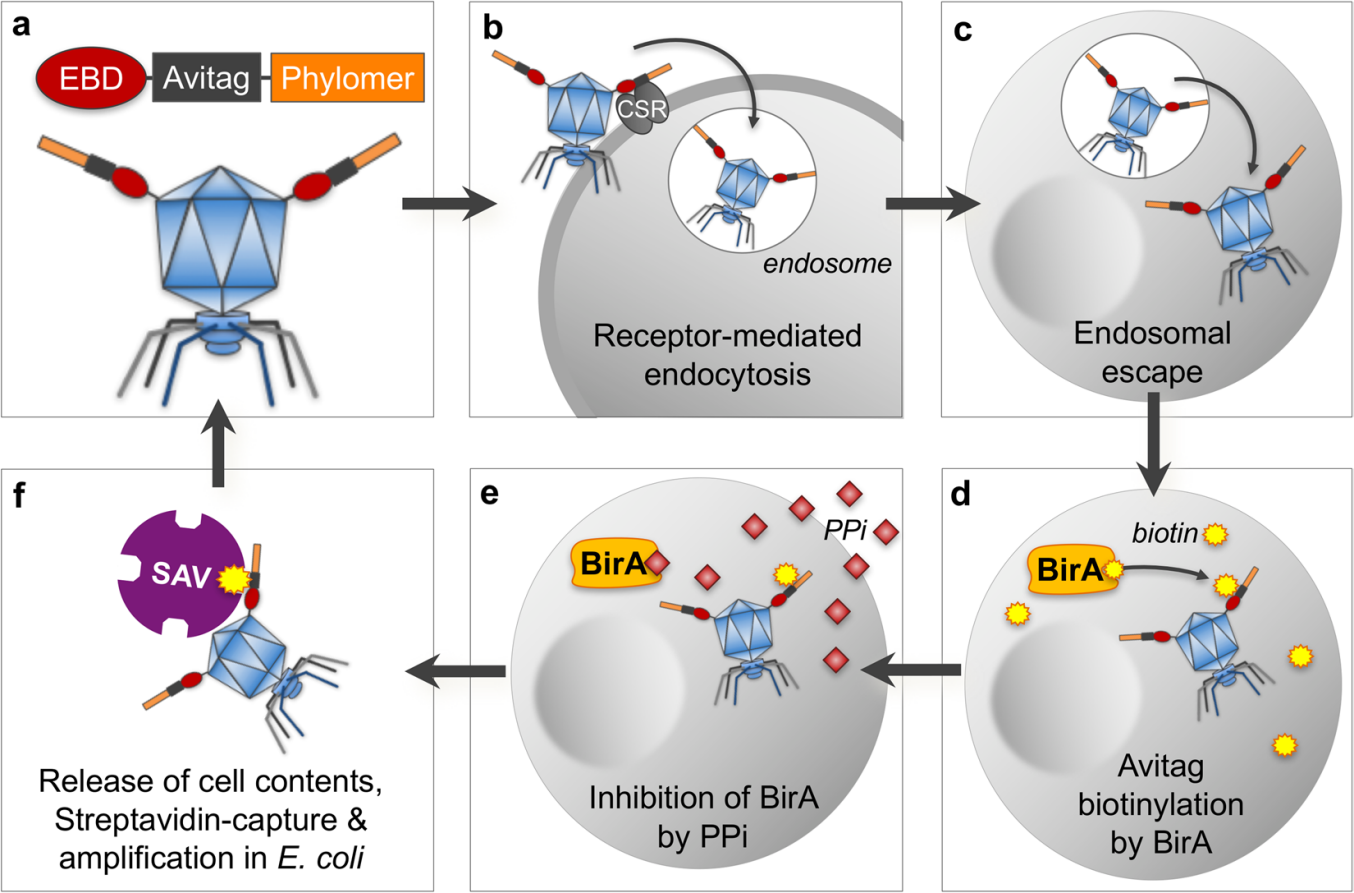
CPP screening and selection process. (**a**) Selection begins with a T7 phage library displaying a fusion of a cargo (in this example, an EGFR receptor binding domain, EBD), an Avitag and Phylomer peptides; (**b**) Avitagged-Phylomer sequences with potential for CPP activity are internalized into cells; this uptake can also be facilitated by binding to specific cell types via a cell surface receptor (CSR) and uptake into endosomes by receptor-mediated endocytosis; (**c**) peptides with capacity for cytosolic delivery allow the phage to enter the cytoplasm; (**d**) selection is performed in mammalian cells expressing the bacterial biotin ligase BirA in the cytoplasm, which ligates free biotin to the lysine residue of the phage-displayed Avitag sequence; this step produces selectively labeled T7 phage that have internalized into the cytoplasm by virtue of the CPP; (**e**) sodium pyrophosphate (PPi), a specific inhibitor of BirA, is added to the cells to terminate the biotinylation reaction; (**f**) cells are lysed and streptavidin-coated magnetic beads (SAV) are added to the lysate to selectively capture and concentrate biotinylated T7 phage. Enriched phage are then amplified in *E*. *coli* and subjected to further rounds of selection. Identification of specific CPPs is achieved by deep sequencing of early selection rounds or by Sanger-sequencing of individual phage clones after 3–4 rounds of selection.

We have developed a phage-based screening platform (Fig. 1) to identify CPPs that internalize and enter the cellular cytoplasm. In a screen for CPPs, mammalian cells expressing biotin ligase (BirA)^33^ are exposed to a Phylomer peptide library, where the Phylomers are expressed on T7 phage as fusions to the Avitag peptide sequence. Phage constructs may include receptor binding domains (e.g., an EGFR-binding domain, EBD), but such targeting sequences are not mandatory for intracellular uptake (Fig. 1a). Avitagged-Phylomer sequences with potential for CPP activity are internalized into cells; this uptake can also be facilitated by binding to a cell surface receptor (CSR) (Fig. 1b). Upon intracellular uptake and cytosolic delivery (Fig. 1c), these CPPs are biotinylated inside cells stably expressing BirA (Fig. 1d).

Cytoplasmic biotinylation modifies the Avitagged phage-displayed CPPs. This biotinylation differentiates the selectants from peptides failing to penetrate the cell or escape endosomes. Before cell lysis, BirA activity is inhibited using sodium pyrophosphate (PPi) (Fig. 1e). This step inactivates BirA to prevent biotinylation of non-internalized Avitagged phage by enzyme released during lysis of the cells. After lysis, internalized biotinylated phage are released and captured by streptavidin-coated magnetic beads (SAV) (Fig. 1f). Sequential screening rounds can then enrich for potent CPP sequences in the released phage population. Individual CPPs are identified by deep sequencing of phage pools from selection rounds or by Sanger sequencing of individual clones.

We first validated the technical foundations of the platform. BirA could successfully biotinylate Avitagged-T7 phage in both *E*. *coli* (Supplementary Fig. 1a) and in mammalian cells and cell lysates (Supplementary Fig. 1b). During phage production, the *E*. *coli* host strain for the phage was transformed with a “decoy” plasmid overexpressing three concatenated Avitag sequences. The resultant decoy protein, an Avitag-trimer, can then outcompete the phage-displayed Avitag sequence for biotinylation by endogenous, bacterial BirA. Expression of the decoy significantly reduced biotinylation of Avitagged-T7 phage or phage libraries that would otherwise occur (Supplementary Fig. 1c,d). After incubation of mammalian cells with the phage-displayed library, PPi was added to inactivate the BirA enzyme (Supplementary Fig. 1e). The reduced background biotinylation is critical to the success of the CPP discovery platform as it removes false positives from the screen.

For proof-of-concept, functional cytoplasmic expression of BirA in mammalian cells was assayed. Biotinylation of a transiently expressed Avitagged-protein in the presence of supplemental biotin was demonstrated in HEK-293/BirA cells engineered for CPP screening (Supplementary Fig. 2a). Thus, stably expressed BirA is capable of biotinylating proteins when the enzyme is expressed inside mammalian cells. BirA expression in the HEK-293/BirA cells was confirmed to be solely cytoplasmic (Supplementary Fig. 2b). In contrast, expression of the late-endosomal marker protein Rab7^34^ was only detected in the membrane-associated cell extract fractions. Having technically validated the underlying principles, we used the platform to discover novel CPP sequences.

CPP screens have two phases: the selection of novel CPPs through iterative rounds of selection followed by characterization of enriched CPPs through sequencing and bioinformatics analysis. Here, ten independent screens were performed using various combinations of Phylomer libraries in three cell lines that were engineered to overexpress BirA. Two cell lines expressed high levels of EGFR (human squamous carcinoma cell line A431, and HEK-293/EGFR stable cell line) and one cell line (HEK-293) expressed relatively low levels (Supplementary Fig. 3). Phylomer libraries were constructed from fragmented genomic material from pathogenic bacteria, archaea and pathogenic viruses (Supplementary Tables 1–3). The libraries were displayed on the surface of T7 phage as fusions to the EBD protein. The EBD sequence served both as a targeting motif and as a model cargo for delivery. Clones from selection rounds 2 through 5 were sequenced.

Analysis of 1363 peptides of >6 aa identified 805 unique sequences that were characterized by an overall increase in peptide length and charge, along with reduced hydrophobicity, compared to the naïve libraries. Clustering analysis identified groups of overlapping hits; the largest cluster consisted of 113 unique sequences derived from a structural polyprotein of the *Sindbis* virus. Importantly, identical or overlapping sequences were identified both as Phylomers enriched during screens and between independent selections. Such non-random enrichment from the same and even different cell lines suggests a sequence of broad CPP activity. A subset of peptide sequences were selected for protein expression. Selection criteria included appearance in multiple screens, identical or overlapping sequences, and suitability for future chemical synthesis (≤45 aa).

**Figure 2:**
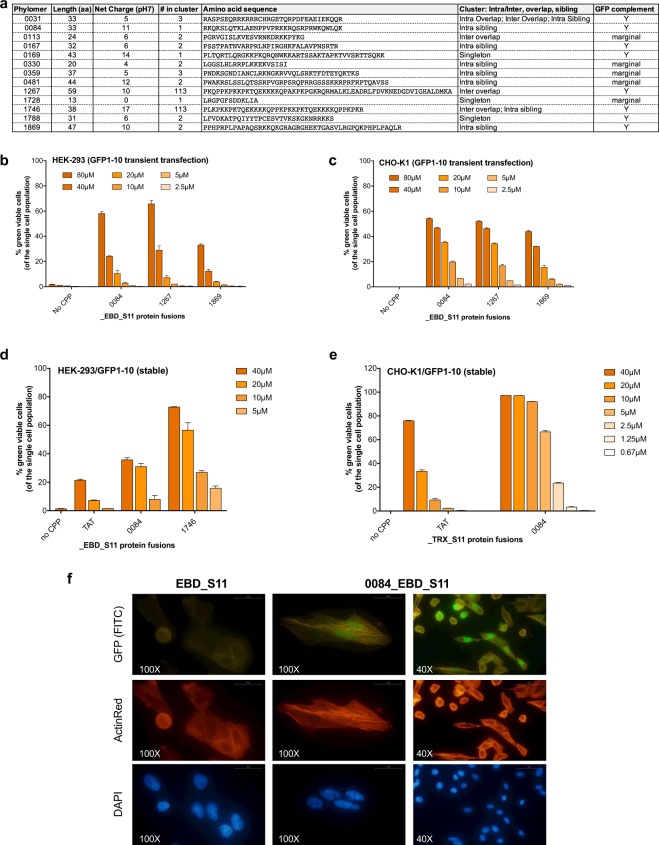
Uptake of Phylomer CPP_EBD_S11 fusion proteins validated by GFP complementation. (**a**) Thirteen Phylomer peptides showed a positive GFP complementation signal, evidence of intracellular delivery. These functionally validated CPPs were of various sizes, net charges and origin. (**b,c**) Dose-dependent uptake of recombinant Phylomer CPP_EBD_S11 fusion proteins was determined by GFP complementation in (**b**) HEK-293 or (**c**) CHO-K1 cells transiently transfected with a hGFP1–10 expressing plasmid. (**d,e**) Dose-dependent uptake of recombinant CPP fusion proteins was confirmed in stable cell lines by GFP complementation of (**d**) EBD_S11 proteins in HEK-293/GFP1–10 and (**e**) TRX_S11 proteins in CHO-K1/GFP1–10 where hGFP1–10 is stably expressed. The greater sensitivity of the stable cell lines enables comparison of uptake at low-dose concentrations. “No CPP” control is shown at the highest concentration (40 µM). In flow cytometry experiments GFP complementation is measured as % fluorescent cells. “No CPP” control is EBD_S11 (b-d) or TRX_S11 (e) protein with no CPP moiety. Results are representative of two independent experiments. Error bars represent standard deviation from the mean between duplicates. (**f**) Fluorescence microscopy visually confirms the CPP-dependent GFP complementation (FITC channel) in CHO-K1 cells transiently transfected with GFP1–10, comparing 0084_EBD_S11 fusion protein (10 µM) to the “No CPP” _EBD_S11 control protein (10 µM). Cells are counter-stained for endogenous cytoplasmic β-Actin (TRITC) and nuclei (DAPI); bar scale is 25 µm.

Thirty-nine candidates were expressed as fusion proteins and screened in a GFP complementation assay^35^. The Phylomers were fused to the S11 split GFP peptide and the EBD used earlier (CPP_EBD_S11 fusion proteins). In this assay, the fluorescent signal is dependent on cytoplasmic delivery of the S11-fused protein. HCC-827 cells, which are positive for human EGFR expression (Supplementary Fig. 3), were transiently transfected with the split GFP complement to the S11 peptide, GFP1–10 plasmid. The following day, cells were treated with the Phylomer CPP_EBD_S11 fusion proteins (10 µM). Thirteen Phylomer fusions showed positive GFP complementation indicating successful cytoplasmic delivery of the protein, and these Phylomer were deemed to be CPPs. Eight Phylomers were strongly positive in this assay (Fig. 2a).

Principal component analysis (PCA) of the sequences for these Phylomer CPPs uncovered sequence clustering into a defined group distinct from both the naïve library (Supplementary Fig. 4a,b) and established, conventional CPPs (Supplementary Fig. 4a,c). Analysis of the biophysical characteristics indicated that Phylomer CPPs were characterized by increased peptide length (p < 0.0001, Supplementary Fig. 5a), increased charge (p = 0.0029, Supplementary Fig. 5b), and reduced hydrophobicity (p = 0.01, Supplementary Fig. 5c) compared to conventional CPPs. Comparison of average amino acid compositions showed significant increases in the number of lysine (K, p = 0.021), proline (P, p = 0.006), glutamine (Q, p = 0.006) and serine (S, p = 0.014) residues for Phylomer CPPs (Supplementary Fig. 5d). The marked increase in lysine residues is a key factor in the high positive net charge of this Phylomer CPP group.

The three most potent Phylomer CPPs (0084, 1267, 1869) were examined further in a dose-dependent split GFP complementation assay. For the three sequences, cytoplasmic delivery and consequent GFP complementation was dose-dependent in both HEK-293 and CHO-K1 cells. As before, such cells were transiently transfected with GFP1–10 and treated with Phylomer_EBD_S11 fusion proteins (Fig. 2b,c). GFP complementation was measured, using flow cytometry, as a percentage of fluorescent cells.

To improve assay sensitivity, Phylomer CPP-driven internalization was then measured in GFP1–10 stable cells. A shorter variant of 1267 (1746) also induced strong, dose-dependent GFP complementation (Fig. 2d). Both CPPs 0084 and 1746 showed superior fluorescence signal and cytoplasmic delivery compared to TAT in HEK-293/GFP1-10 cells. CPP 0084 was also expressed as a Thioredoxin_S11 fusion protein (0084_TRX_S11) to demonstrate delivery of another, independent protein cargo. Uptake of 0084_TRX_S11 was reproducibly delivered to the cytoplasm with the limit of detection as low as 1.25 µM in CHO-K1/GFP1-10 cells (Fig. 2e); this cell line is negative for expression of human EGFR (shown in Supplementary Fig. 3). Thus, the observed CPP activity was not dependent on the presence of or binding to hEGFR. In contrast, the TAT complementation signal was not detected at these lower concentrations.

Fluorescence microscopy was used to visually confirm the GFP flow cytometry signal. GFP complementation was observed in CHO-K1 cells transiently transfected with GFP1–10 and treated with 0084_EBD_S11 (Fig. 2f). Treatment with EBD_S11 alone showed no background signal, indicating that a CPP moiety is necessary for internalization and GFP complementation in these cells. Cells were counter-stained for endogenous cytoplasmic β-Actin (TRITC) and nuclei (DAPI) to visualize their cell architecture and verify cell integrity.

Comparison of circular dichroism (CD) spectra showed the Phylomer CPPs (0084, 1746, 1869) with the highest GFP-complementation signal were largely random coiled and not highly structured in solution. The CD spectra of Phylomer CPPs display unique and different conformational characteristics at different pH and in the presence of SDS micelles simulating membrane environments (Supplementary Fig. 6). To functionally validate the platform we selected a lead candidate for more detailed assessment. CPP 1746 fulfilled multiple desirable criteria: the sequence was identified in multiple selections, was a member of the large *Sindbis* sequence cluster, showed excellent uptake in GFP complementation assays, and was amenable to synthesis. For all these reasons, 1746 was selected as the lead peptide for this validation and further studies.

### Screen-enriched sequence clusters guide optimization

**Figure 3:**
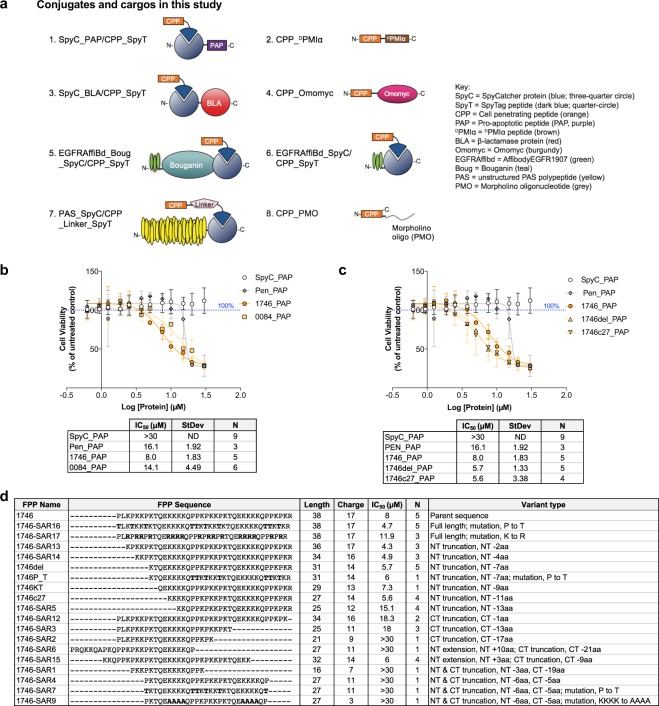
Initial optimization of a Phylomer CPP sequence using cluster alignments and a PAP-based viability bioassay. **(a)** Diagrams of SpyTag (SpyT)/SpyCatcher (SpyC) fusion protein conjugates and other cargos used in this study. In protein ligation^36^ a short peptide sequence, SpyTag (SpyT), forms an isopeptide bond with the SpyCatcher (SpyC) partner protein in an irreversible peptide-protein coupling. **(b)** SpyC_PAP/CPP_SpyT conjugate uptake has a dose dependent decrease in CHO-K1 cell viability for Phylomer and Penetratin-delivered protein, assessed by resazurin reduction potential. The 1746_SpyT/SpyC_PAP conjugate (1746_PAP) showed greatest potency, followed by 0084_SpyT/SpyC_PAP conjugate (0084_PAP). Penetratin_SpyT/SpyC_PAP conjugate (Pen_PAP) is included as a positive control for the assay. SpyC_PAP treatment showed no activity at all concentrations tested up to 30 µM. Calculated IC_50_ values are presented below the graph. Data are from *N* independent experiments. Error bars represent standard deviation from the mean. Significance was assessed by one-way ANOVA. **(c)** Uptake of 1746_PAP and potent variants with reduced charge conjugated to SpyC_PAP (1746del_PAP and 1746c27_PAP) show dose-dependent decrease in CHO-K1 cell viability, assessed by resazurin reduction potential. Delivery using 1746del and 1746c27 variants showed improved, lower IC_50_ compared to 1746. Calculated IC_50_ values are shown below the graph. Data are from *N* independent experiments. Error bars represent standard deviation from the mean. Significance was assessed by one-way ANOVA. **(d)** Summary of 1746 variant testing and selection. Parameters include peptide length, sequence charge, IC_50_ in viability assays where 1746_SpyT variants deliver conjugated SpyC_PAP into CHO-K1 cells. CPP derivatives are aligned to the 1746 parental sequence with an explanation of variant type. N is the number of independent PAP assays in which each sequence was assessed.

Phylomer CPP optimization focused on two goals. First, we wished to identify the minimal peptide domain without compromising functional activity, and second, other modifications to increase potency were assessed. A modular approach using the SpyCatcher (SpyC)/SpyTag (SpyT) protein ligation technology^36^ was applied to facilitate this. The SpyT peptide sequence binds to and forms a covalent, isopeptide bond with the SpyC protein. Thus, by synthesizing CPP sequences fused to the SpyT sequence, the CPP-SpyT peptides can be coupled to any SpyC-fused cargo. Figure 3a and supplementary material (Supplementary Tables 4–8) provides diagrams and information on the conjugates and other cargos used in this study.

The potency of each conjugate guided optimization, and conjugates were assessed in functional assays where readout is dependent on cytosolic delivery of a peptide cargo. Intracellular delivery of the proapoptotic peptide PAP^37^ has been shown to directly induce dose-dependent apoptotic cell death^38^. Each Phylomer CPP was synthesized as a SpyTag fusion (CPP_SpyT) and was then conjugated to a SpyC_PAP fusion protein. CHO-K1 cells were treated with the resultant conjugates (Fig. 3a, cargo 1). A conventional CPP conjugated to PAP, Penetratin_SpyT/SpyC_PAP, was included as a positive control for the assay. Cell death induced by 1746_SpyT/SpyC_PAP (IC_50_ = 8.0 µM) was significantly greater (p = 0.01) than cell death from Penetratin-mediated (IC_50_ = 16.1 µM) delivery of SpyC_PAP protein (Fig. 3b). As expected, the unconjugated SpyC_PAP negative control showed no effect.

To reduce the size and charge of 1746, a range of sequence variants were designed. These included N- and C-terminal truncations, amino acid (aa) substitutions, and two N-terminal sequence extensions based on longer sequences from the 1746 sequence cluster (Supplementary Table 6 lists sequences for these variants). The effect of these modifications on CPP potency was assessed in the PAP assay and compared to unmodified 1746 (Fig. 3c,d). Overall, the potency was retained or improved in variants with charge ≥ 13. N-terminal truncation by up to 7 aa (1746del, IC_50_ = 5.7 µM) or 11 aa (1746c27, IC_50_ = 5.7 µM) also had improved CPP potency (p = 0.001 and p = 0.002, respectively) compared to Penetratin. However, a 13 aa N-terminal reduction (1746-SAR5) showed a 3-fold loss of activity. C-terminal truncations were detrimental, with even a single amino acid deletion (1746-SAR12) showing 2.2-fold reduction in potency.

Substituting different amino acids into the CPP sequence also affected potency. Mutating proline residues to threonine improved 1746 activity by 1.7-fold (1746-SAR16), whereas activity was unaffected for the N-terminal 7 aa truncated variant (1746P_T). Mutating lysine residues to arginine (1746-SAR17) resulted in a 1.5-fold reduction in potency. N-terminal extension (1746-SAR6) did not compensate for the decrease in potency due to a C-terminal truncation in the same variant. Other truncation variants showed no activity in the PAP assay. In summary, the data demonstrate that the C-terminal arginine residue and the lysine residues are vital for full CPP activity, C-terminal truncation is detrimental, and proline residues are not essential. The data also identified the C-terminal 27 residues (1746c27) as the minimum domain sufficient for potent activity. The 1746c27 variant was also largely random coiled in solution, similar to full-length 1746 peptide (Supplementary Fig. 6).

### Phylomer CPPs show minimal evidence of toxicity

**Figure 4:**
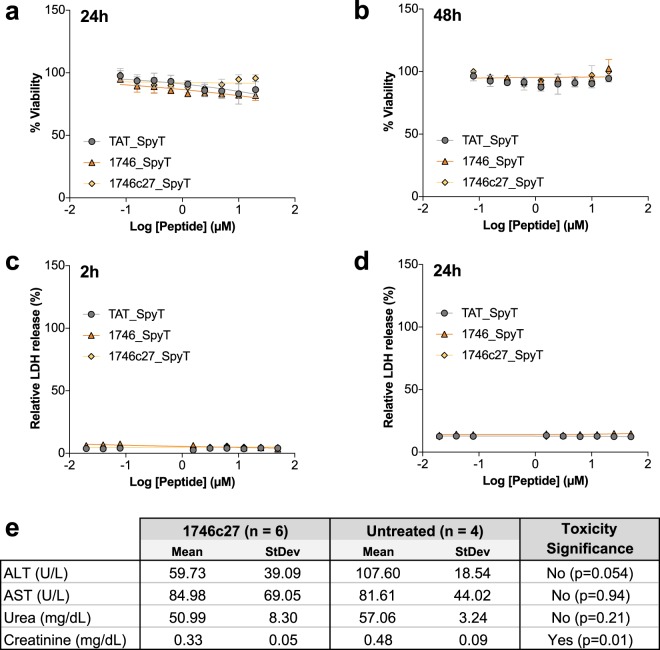
Phylomer CPPs show minimal toxicity. Phylomer CPP toxicity was assessed in cells and in mice. (**a**,**b**) Cell viability effect on CHO-K1 cells of 1746 and 1746c27, alongside TAT, following 24 h (**a**) and 48 h (**b**) incubation with peptides. Cell viability was assessed by resazurin reduction potential. (**c**,**d**) Membrane integrity of CHO-K1 cells were assessed by LDH release following 2 h (**c**) and 24 h (**d**) incubation with peptides. All CPPs remained non-toxic to cells. Results are representative of three independent experiments. Error bars represent standard deviation from the mean of triplicate samples. (**e**) 1746c27 toxicity *in vivo* was assessed by measuring the urea, creatinine, aspartate transaminase (AST) and alanine transaminase (ALT) concentrations in serum from mice (n = 6) treated with daily IP injections of 1746c27 for 7 days based on EMA- and FDA-approved standard preliminary toxicity testing guidelines. Minimal evidence of toxicity was seen following treatment. AST and urea levels showed no significant difference compared to untreated control mice (p = 0.94 and p = 0.21, respectively). ALT levels showed no significant difference and were lower compared to untreated control mice (p = 0.054), and creatinine levels were significantly lower compared to untreated mice (p = 0.01), which supports the finding of minimal toxicity. Significance was assessed by unpaired, two-tailed T-test.

To ensure that 1746 and 1746c27 are not innately cytotoxic, we assessed their effect on cell viability. CHO-K1 cells were treated with CPPs in the presence of sera at 24 h (Fig. 4a) and 48 h (Fig. 4b). The effects of CPPs on cellular membrane stability were assessed using a LDH enzymatic assay, measuring LDH release at 2 h (Fig. 4c) and 24 h (Fig. 4d) after peptide addition. All CPPs showed no obvious cytotoxic activity up to the highest concentrations tested (50 µM). Since 1746c27 combined strong activity, reduction in length, reduction in charge and no measurable cytotoxicity, it was selected as our lead CPP.

To examine 1746c27 toxicity *in vivo*, a cohort of adult male C57BL/6J mice (n = 6) were treated with the CPP. Following approved standard preliminary toxicity testing guidelines (Food and Drug Administration, FDA, and European Medicines Agency, EMA), mice were injected intraperitoneally daily for 7 days with 40 mg/kg of 1746c27. After a further 8 days the mice were culled (on day 15) and serum was extracted and used to examine kidney and liver toxicity. Urea, creatinine, aspartate transaminase (AST) and alanine transaminase (ALT) concentrations were measured. We found minimal evidence of toxicity following treatment of the mice (Fig. 4e). AST (p = 0.94) and urea (p = 0.21) levels showed no significant difference compared to untreated control mice. ALT (p = 0.054) and creatinine (p = 0.01) levels were lower compared to untreated control mice, which supports the finding of minimal toxicity.

### Phylomer CPPs show potent delivery of a range of biologics

**Figure 5:**
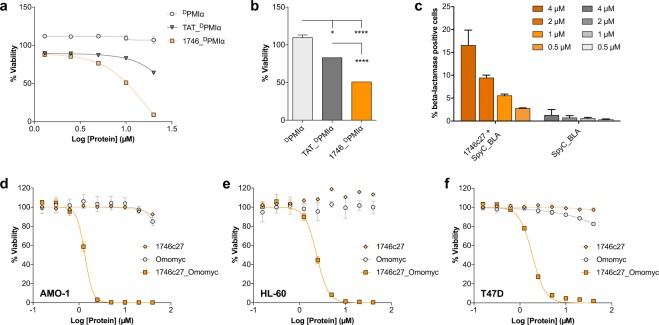
CPP-mediated intracellular delivery of a range of cargos into cells. (**a**) Uptake of ^D^PMIα peptide into cells shows a dose dependent decrease in T47D cell viability when ^D^PMIα was delivered by 1746, and to a lesser extent, by TAT. Cell viability was assessed by resazurin reduction potential after 48 h incubation with peptides. (**b**) Comparison of 10 µM peptide treatments shows CPP_^D^PMIα peptides are significantly more potent compared to ^D^PMIα peptide alone. 1746_^D^PMIα treatment also shows significantly reduced cell viability compared to TAT. Results are representative of 3 independent experiments. Error bars represent standard deviation from the mean of duplicate samples. Significance was assessed by one-way ANOVA with Dunnett’s multiple comparison test (*p < 0.05; ***p < 0.001; ****p < 0.0001). (**c**) β-lactamase flow cytometry assay shows dose-dependent cell entry of SpyC_BLA/1746c27_SpyT conjugates at concentrations with the limit of detection as low as 500 nM in CHO-K1 cells; no marked signal is observed for the control protein SpyC_BLA. Data shown are from two independent experiments. Error bars represent standard deviation from the mean. (**d–f**) Treatment of (**d**) AMO-1 (plasmacytoma), (**e**) HL-60 (promyelocytic leukemia), and (**f**) T47D (breast cancer) cell lines with 1746c27_Omomyc protein. After 48 h incubation, cell viability was assessed by measuring ATP activity. Results show strong, similar efficacy of 1746c27_Omomyc across all three cell lines. The average IC_50_ values for 1746c27_Omomyc treatment in the three cell lines were calculated as 1.28 µM (AMO-1), 1.88 µM (HL-60) and 1.67 µM (T47D). Peptide 1746c27 alone shows no significant cytotoxicity. Control protein Omomyc exhibits a minor effect on cell viability only at the highest concentrations (mid to high micromolar potencies). Results are representative of two independent experiments. Error bars represent standard deviation from the mean of duplicate samples.

A variety of cell types and cargos confirmed the potency and versatility of Phylomer CPP delivery. Different functional assays applied readouts designed to directly quantify delivery of an established biologic cargo. First, we showed successful Phylomer CPP delivery of small biologic cargo using ^D^PMIα^39^ peptide. When fused to CPPs, pro-apoptotic ^D^PMIα has been shown to internalize, directly bind to MDM2 and lift p53 suppression, thus causing cytotoxicity in cancer cells overexpressing MDM2^39^. CPP_^D^PMIα synthetic peptides (Supplementary Table 7 and Fig. 3a, cargo 2) reduced viability of human breast tumor cells (T47D) in the presence of sera, indicating the successful delivery of ^D^PMIα into the cells (Fig. 5a). Treatment with peptide 1746_^D^PMIα (IC_50_ = 9.1 µM) was significantly more cytotoxic than ^D^PMIα alone, and showed greater potency than TAT_^D^PMIα (IC_50_ = 36.6 µM), which was included as a positive control in the assay (Fig. 5b).

Second, β-lactamase was used as a model protein cargo, to assess the ability of Phylomer CPPs to deliver functional enzymes into cells^40^. Cytoplasmic β-lactamase activity can be specifically detected in a sensitive, rapid enzymatic assay that measures the hydrolysis of a fluorescent substrate developed for the detection of β-lactamase in mammalian cells^41–44^. Thus, recombinant SpyC_β-lactamase was conjugated to 1746c27_SpyT (Fig. 3a, cargo 3). Its successful internalization was detected in a dose-dependent manner, with a limit of detection of 500 nM in CHO-K1 cells in the presence of sera (Fig. 5c). Confocal live cell fluorescence microscopy visually confirmed the uptake of 1746c27_SpyT/SpyC_β-lactamase in T47D cells, a second and independent cell type for this assay. Internalization of functional β-lactamase and subsequent substrate cleavage was dose-dependent and observed at all concentrations (2 µM, 4 µM, 8 µM; Supplementary Fig. 7a,c,e, respectively). Internalization of unconjugated SpyC_β-lactamase was not observed at any of the same concentrations (Supplementary Fig. 7b,d,f, respectively). These experiments demonstrated both the delivery of β-lactamase and its continued functionality once inside the cell.

Next 1746c27 was used to deliver the Omomyc^45,46^ protein cargo. This well-characterized dominant-negative protein directly binds and inhibits the transcription factor MYC. A master regulator of critical cellular processes^47^, MYC is an intracellular oncoprotein target deemed “undruggable” using conventional biological therapeutics. Treatment with recombinant 1746c27_Omomyc (Fig. 3a, cargo 4) induced a dose-dependent decrease in cell viability in MYC-dependent blood cancer cell lines and T47D cells in the presence of sera. This intracellular therapeutic was particularly potent, with average IC_50_ values of 1.28 µM (AMO-1, Fig. 5d), 1.88 µM (HL-60, Fig. 5e) and 1.67 µM (T47D, Fig. 5f). Complete cell death was observed at concentrations ≥5 µM (AMO-1) or ≥10 µM (HL-60 and T47D). The potency of 1746c27_Omomyc was greater than small molecule inhibitors of MYC, 10058-F4 and KJ-Pyr9 (Supplementary Fig. 8). In contrast, Omomyc alone did not affect cell viability, with the exception of a slight reduction in cell viability for AMO-1 and T47D cells at doses above 10 µM. Treatment with 1746c27 peptide alone showed no notable effect on cell viability in these assays.

### Compatibility with targeting and half-life extension methods

**Figure 6:**
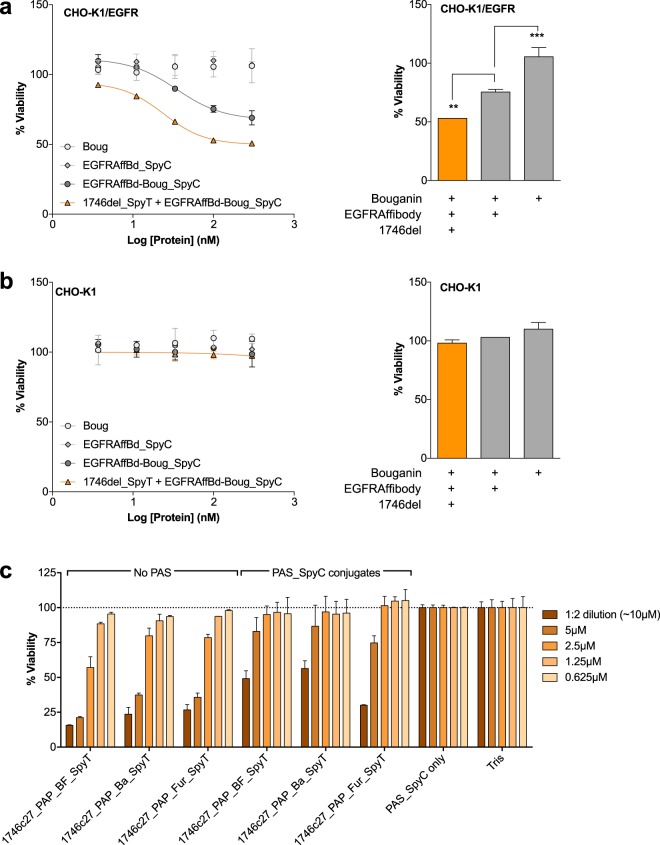
Phylomer CPP delivery is compatible with cell specific targeting and half-life extension approaches. (**a**) CHO-K1 cells stably expressing EGFR receptor or (**b**) CHO-K1 cells were treated with 1746del_SpyT conjugated to EGFRAffibody_Bouganin_SpyC (EGFRAffbd_Boug_SpyC) toxin. After 48 h incubation, the cell viability was assessed according to the resazurin reduction potential. Comparison of 100 nM immunotoxin treatment in CHO-K1_EGFR (**a**, right) vs CHO-K1 (**b**, right) cells shows that conjugation to 1746del improved delivery compared to the EGFRAffibody alone, and that the 1746del-conjugate retains EGFRAffibody-encoded specificity. Results are representative of 3 independent experiments. Error bars represent standard deviation from the mean of duplicate samples. Significance was assessed by one-way ANOVA with Dunnett’s multiple comparison test (**p < 0.01; ***p < 0.001). (**c**) T47D cells were treated with 1746c27_PAP_linker_SpyT with and without conjugation to the PAS_SpyC fusion protein. 1746c27-dependent PAP-induced cytotoxicity was detected for all PAS conjugates in comparison to the buffer control (Tris). The Furin-cleavable conjugate exhibited the greatest potency, but all PAS conjugates showed dose-dependent cell toxicity in a comparable concentration range. Linkers are Cathepsin B FKFL cleavage motif (BF), Cathepsin B Valine-Citrulline cleavage motif (Ba) and Furin RKKR cleavage motif (Fur). Results are representative of two independent experiments. Error bars represent standard deviation from the mean of duplicate samples.

To demonstrate the compatibility of a Phylomer CPP with cell targeting approaches, we fused a well-characterized targeting domain (Affibody_EGFR-1907_^48^ that binds human EGFR) to a potent immunotoxin (Bouganin)^49,50^. Delivery of the CPP fusion immunotoxin (Fig. 3a, cargo 5) was assessed in matched CHO-K1 cell lines (±hEGFR receptor). Delivery by the Phylomer CPP improved the potency of the Affibody_Bouganin cargo by 46%, comparing IC_50_ values of 24 nM (1746del_SpyT + EGFRAffbd_Bouganin_SpyC) and 35 nM (EGFRAffbd_Bouganin_SpyC alone). In contrast, Bouganin and Affibody_SpyC (Fig. 3a, cargo 6) proteins alone had no effect on either cell line at the concentrations tested. Importantly, at these concentrations, the CPP-delivered toxin was highly potent in hEGFR-positive cells (Fig. 6a) and not in hEGFR-negative cells (Fig. 6b). This experiment showed that the Phylomer CPP enhanced the delivery of EGFRAffbd_Bouganin_SpyC and retained the Affibody-mediated cell specificity at the doses tested.

The compatibility of Phylomer CPPs and plasma half-life extension technology was evaluated with PASylation as a standard exemplar^51^. The PAP assay examined the potency of 1746c27_PAP_linker_SpyT variants (Supplementary Table 8) conjugated to PAS_SpyC (Supplementary Table 5). The PAS polypeptide adopts a random-coil conformation with an increased hydrodynamic volume and retards renal filtration, thus extending the half-life of biologics in a tunable manner^51^. CPP conjugates (Fig. 3a, cargo 7) were applied to T47D cells in the presence of sera and their potency compared. While there was some decrease in potency from conjugation to the PAS domain, CPP-dependent PAP-induced cytotoxicity was still detected for all conjugates (Fig. 6c). The linkers used in this study were the Cathepsin B FKFL cleavage motif (BF), the Cathepsin B Valine-Citrulline cleavage motif (Ba), and the Furin RKKR cleavage motif (Fur). Of these linkers, the furin-cleavable conjugate showed the greatest potency in this assay. This result suggests that cleavage from PAS, for example by endosomal proteases, may be necessary to maximize therapeutic efficiency. Taken together, these proof of concept studies suggest that potent Phylomer CPPs can be engineered for cell specificity, if required, and Phylomer CPPs are amenable to next generation half-life extension technologies.

### Delivering a therapeutic in an *in vivo* disease model of DMD

**Figure 7:**
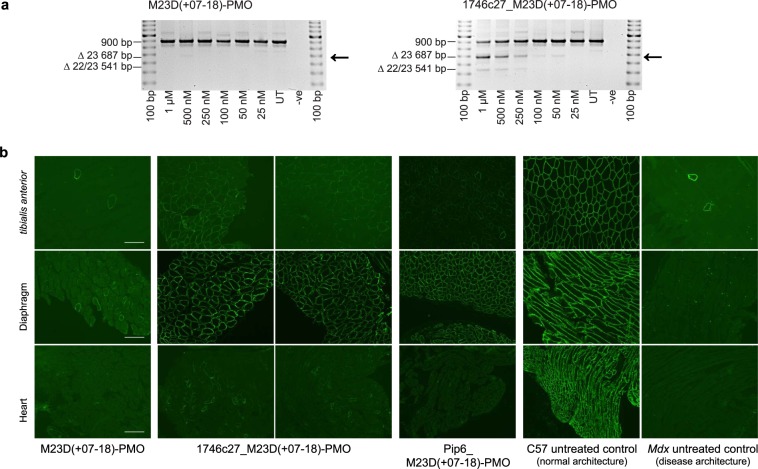
1746c27 delivery of a PMO therapeutic *in vivo* in a disease model of DMD. (**a**) Intracellular delivery of 1746c27_M23D(+7–18) induces dose-dependent skipping of exon 23 of the *dystrophin* gene in differentiated murine *H-2K*^b^*-tsA58* myogenic cultures. Exon skipping (marked by arrow) can be detected by RT-PCR from doses of 50 nM 1746c27_M23D(+7–18), but is not detected at any dose of M23D(+7–18) morpholino alone or the untreated cells (UT). (**b**) Tissue staining for dystrophin expression shows *in vivo* treatment of C57BL/10ScSn^*mdx*^ mice (5 treatments over two weeks, with 4 nmol per dose) of 1746c27_M23D(+7–18) causes improved dystrophin protein levels and muscle architecture in the diaphragm, and to a lesser extent the *tibialis anterior* (samples taken from two independently-treated mice, n = 2). This improvement is compared to untreated C57BL/10ScSn^*mdx*^ mice (Mdx untreated control) or those treated with the M23D(+7–18) morpholino alone (M23D(+7–18)-PMO). Treatment with Pip6-morpholino conjugate (Pip6_M23D(+7–18)-PMO) was a positive control for antisense-induced dystrophin expression. Tissue staining for dystrophin expression in C57BL/10ScSn mice (C57 untreated control) shows normal muscle architecture for comparison. Bar scale is 100 µm.

Finally, we validated the ability of 1746c27 to deliver an oligonucleotide therapeutic in an *in vivo* disease model of Duchenne muscular dystrophy (DMD). The *mdx* mouse is a naturally occurring disease model of dystrophin-negative muscular dystrophy with a well-characterized mutation in exon 23 of the *dystrophin* gene transcript^52^. Phosphorodiamidate morpholino oligomer (PMO) M23D(+7–18)^53^ targets exon 23 of the *dystrophin* gene transcript and induces exon skipping to produce a shorter, yet functional dystrophin protein. To assess Phylomer CPP delivery of this PMO, 1746c27 was conjugated to M23D(+7–18) (Fig. 3a, cargo 8), and transfected into murine *H-2K*^b^*-tsA58* myoblasts *in vitro*. The transfected myoblasts were incubated for 4 days post-transfection in the absence of sera. Exon skipping was detectable at the RNA level when cells were treated with as little as 50 nM of 1746c27_M23D(+7–18) (Fig. 7a).

To assess the *in vivo* potency of 1746c27_M23D(+7–18), C57BL/10ScSn^*mdx*^ mice (initially at 3–5 days of age) were treated with five intra-peritoneal injections of the CPP-PMO cargo over two weeks at 4 nmoles per dose (the injected volume increased with the increased weight of the mice as noted in the Methods). For comparison, Pip6 was also conjugated to M23D(+7–18) and injected into mice; Pip6 is a traditional carrier CPP for peptide-oligonucleotide conjugates in the *mdx* disease model of DMD^54^. Two weeks after the end of treatment, tissue cryosections from mice showed decreases in the disease phenotype from mice treated with 1746c27_M23D(+7–18). Specifically, we observed an increase in dystrophin expression and markedly improved muscle architecture in the diaphragm compared to the untreated mice and those treated with M23D(+7–18) alone (Fig. 7b).

Systemic distribution of 1746c27_M23D(+7–18) is evidenced by global expression of induced dystrophin in distal muscles, such as the *tibialis anterior*. The heart muscle has proved refractory to the uptake of nucleic acid analogue therapeutics, and even the patchy, low-level expression of dystrophin in heart tissue seen in our assays is supporting evidence for an *in vivo* therapeutic effect. A screen of 1746c27_M23D(+7–18) targeting a constitutively expressed transcript supports these findings and relative delivery into the different tissues, using RT-PCR to detect exon skipping (Supplementary Fig. 9a) or immunoblotting for dystrophin protein (Supplementary Fig. 9b). Since the antisense molecule specifically alters exon selection during splicing of the target pre-mRNA sequence, the effects are only evident in cells expressing the target gene protein or mRNA transcript.

## Discussion

The discovery of new and efficient CPPs with potent capacity to deliver biologic cargos for research, diagnostic, and therapeutic applications remains a key challenge. The BirA-based CPP discovery screen presented here is an innovative and generalizable platform that enables selection of CPPs with good cell entry potency, independent of any particular mechanism of internalization. The approach is compatible with any cell line engineered to express BirA. The modular library design offers the potential to engineer specificity into the CPP selection through targeting different cell types or screening with various cargos. This feature is highly desirable as delivery specificity is often considered a major design issue for CPPs^1^. CPP screens are compatible with antibody fragment, small scaffold or generic peptide libraries, allowing screening in the context of a cargo. A key advantage of Phylomer peptide libraries is that they encompass multiple genomes represented at high redundancy. This design allows the identification of overlapping clusters of sequences whose commonalities can guide subsequent maturation strategies, as shown here.

We identified 13 unique CPPs derived from diverse organisms. All could deliver recombinant protein cargo into the cytoplasm of cells. Phylomer CPPs 1746 and 0084 showed greater activity than conventional CPPs, particularly at lower concentrations where uptake is less likely to be due to non-specific flooding into the cytosol^19^. We used clustering analysis and conventional sequence optimization to identify a minimal sequence that retains the strong potency of the parental sequence. This minimal peptide, 1746c27, was compatible with engineering approaches for cell targeting and half-life extension that are often employed to overcome the lack of specificity and the quick clearance typically seen with traditional CPPs^55^. Specifically, the Phylomer CPP retained EGFR-dependent specificity when combined with a targeting Affibody, and also largely maintained potency after PASylation^51^.

Phylomer CPP efficacies were validated with successful delivery of multiple biologically-relevant cargos at low concentrations, rare for CPPs (as reviewed in^56^). In particular, 1746c27 showed strong potency at concentrations as low as sub-micromolar. This highly desirable potency is exceptionally useful for therapeutics; in theory, such activity can avoid high dosage concentrations, and consequently lower toxicity, membrane disruption and the manufacturing costs. We demonstrated successful delivery of recombinant β-lactamase, recombinant PAP and ^D^PMIα peptide into cells with greater potency compared to the conventional CPPs assessed alongside (on average, half the size of 1746c27). Phylomer 0084 also successfully delivered multiple cargos including TRX_S11, recombinant PAP and ^D^PMIα peptide (data not shown). In addition, we used 1746c27 to successfully deliver Omomyc, a well-characterized dominant-negative inhibitor of MYC, demonstrating excellent potencies for this protein-based biological therapeutic (IC_50_ values 1.3–1.9 µM).

Finally, we observed potent delivery in a disease model of muscular dystrophy. The Phylomer CPP 1746c27-delivered PMO cargo *in vivo*, and induced production of functional dystrophin in distal muscles. While the major impact of the absence of dystrophin is on muscle (striated, smooth and cardiac muscles), various dystrophin isoforms are expressed in many tissues. Therefore, global distribution of the cargo as shown here is preferable. The low-level expression of dystrophin in the heart muscle is also encouraging, as cardiac muscle has proved refractory to uptake of nucleic acid analogue therapeutics^54^. As dystrophin levels in excess of 3–5% of normal are expected to confer substantial therapeutic benefit in DMD^57^, this study provides strong evidence for the power of Phylomers to deliver high-potency therapeutics *in vivo*.

In summary, our CPP discovery platform offers a versatile approach to discover functional CPPs that can internalize into cells and delivery biologic cargoes into the cytosol. We successfully identified novel Phylomer CPPs that are potent, versatile, compatible with engineering approaches, amenable to synthesis or conventional recombinant production, and thus compatible with cost-efficient, scaled manufacturing. Further, the leading Phylomer CPPs are largely non-toxic. Importantly, these CPPs can deliver a wide range of biologic cargos ranging from large proteins to smaller peptides and oligonucleotides, both *in vitro* and *in vivo*. The increasing interest in the CRISPR-Cas9 system highlights the need for delivery of both proteins and nucleic acids, as demonstrated here. We propose that the innate delivery efficiency of Phylomer CPPs addresses a key challenge for intracellular macromolecular therapeutics by enabling more biological drug payloads to reach diverse disease targets within the cell.

## Methods

### Library construction

Phylomer libraries were generated^58^ by preparing DNA from genomic sources (Supplementary Tables 1–3) using T7N6 (libraries T08 and T09; 5′-GTAATACGACTCATACAATTGCNNNNNN-3′) or T7N12 (viral library T12; 5′-GTAATACGACTCATACAATTGCNNNNNNNNNNNN-3′) random amplification primers. Amplified fragments were cloned into a T7Selected10–3b-based vector (T7mid-EBD-HA-Avi) 3′ of the EBD_HA_Avi fusion sequence at a 1:1 (T8, T9) or 1:1.6 (T12) vector-to-insert ratio using Novagen’s T7Select Phage Display System (Merck KGaA) and *E*. *coli* BLT5615 host strain, which was pre-transformed with the pSumo_Avi3 construct. T7mid-EBD-HA-Avi vector was produced with a Lambda DNA Purification Kit (Agilent) and T7Select Phage Display System (Novagen). The vector was prepared for cloning by digestion with *EcoR*I-HF (NEB, 10 U/μg) for 2 h at 37 °C, heat inactivated at 65 °C for 5 min; dephosphorylated with Antarctic Phosphatase (NEB, 2 U/μg) for 20 min at 37 °C, and heat inactivated at 65 °C for 20 min.

The T12 viral sequences were created from selected viral proteins and synthesized in pUC57-Kan (Genscript), codon optimizing for both expression in *E*. *coli* and removal of *BamH*I and *Mfe*I restriction sites. Sequences were amplified by PCR (94 °C/2 min; then 30 cycles of: 94 °C/30 sec, 62 °C/30 sec, 68 °C/3 min; then 68 °C/10 min) using universal flanking primers (5′-CTCGGTACCTCGCGAATGC-3′; 5′-CAGGCCTCTGCAGTCGACG-3′; Sigma Aldrich) and Platinum PCR SuperMix (Life Technologies) in the presence of 5% DMSO and 1 M betaine. PCR products were gel-purified with a 1% agarose gel stained with SYBR-Safe (Life Technologies) using a QIAquick Gel Extraction Kit (QIAGEN), and quantified by Qubit BR assay (Life Technologies). Then 1 μg purified DNA was digested with 10 U *BamH*I (Promega) for 1 h at 37 °C, heat inactivated at 70 °C for 10 min, and purified using a QIAquick PCR purification kit (QIAGEN) and quantitated by Qubit BR assay (Life Technologies). 40 ng of each digested and purified viral DNA fragment was used as a template for Phylomer generation.

### Plasmids

For recombinant protein expression and pSUMO_Avi3 constructs, sequences were optimized for *E*. *coli* codon usage and synthesized (ATUM; see Supplementary Tables 4–5 and 9 for details of expressed sequences). The insert for pSUMO_Avi3 was then sub-cloned into an expression plasmid with chloramphenicol selection using standard cloning techniques. Plasmid stocks were prepared using Plasmid Plus DNA kits (QIAGEN).

### CPP screening

Briefly, for a phage screen, adherent HEK-293/BirA cells were seeded into T25 flasks (1.5 × 10^6^ cells/flask) and incubated overnight. In a selection round, the cell media was removed and cells were washed with warm complete media (antibiotic free) before addition of D-biotin (1 µM final concentration) and approximately 2 × 10^10^ pfu of PEG-purified, streptavidin-cleared T7 phage. Phage and cells were incubated for 1 h at 37 °C with 5% CO_2_, before cells were washed with warm DMEM. Phage bound to the cell surface were rendered non-infective by addition of 1 ml acidified DMEM (pH 2) to the flask, which was passed over the cell surface before immediately washing three times with complete DMEM medium to completely remove acid. Internalized phage were recovered by lysing cells on flask with 400 µl M-PER reagent (Thermo Fisher Scientific) containing 1 mM sodium pyrophosphate (as a BirA inhibitor, Sigma Aldrich). Insoluble cell debris was removed by centrifugation and Avitagged phage in the supernatant was recovered by binding to M280 streptavidin Dyna beads (50 µl) with rotation for 30 min. Samples were washed four times with PBS/0.05% Tween-20, resuspended in PBS and treated with 0.25% trypsin-EDTA solution for 5 min to elute phage from beads. After removal of a small aliquot for phage titration, the remaining sample was used to infect exponentially growing *E*. *coli* 5615 co-expressing pSumo_Avi3 (Supplementary Table 9) for amplification of T7 phage.

Phage clones were identified by extracting T7 DNA from agar plugs and amplifying the insert by PCR using universal flanking primers (5′-GCAATGGGCCACGGTGGTCTTCGC-3′; 5′-ACCCCTCAAGACCCGTTTAGAG-3′; Sigma Aldrich). PCR-amplified inserts were sent for Sanger sequencing (BDT v3.1) before bioinformatics analysis to determine Phylomer sequences.

**Screens summary**

**Table:** 

**Cell line**	**Library**	**Sequenced peptides from rounds**
HEK-293/BirA	T08	3–5
HEK-293/BirA	T09	3–5
HEK-293/BirA	T08 + T09	3–4
A431 (rounds 1–2), HEK-293/BirA (rounds 3–5)	T08 + T09	4–5
A431/BirA polyclonal	T08 + T09	2–5
A431/BirA	T08 + T09	2–4
A431/BirA	T09 + T12	3–4
A431/BirA	T09 + T12	3–4
HEK-293/EGFR-BirA	T09 + T12	3–4

### Mammalian cell culture

HEK-293, A431, CHO-K1 cell lines were obtained from ATCC. These cell lines, HL-60 and AMO-1 cell lines (Dr. D Fairlie, Olivia Newton John Cancer Research Institute), and T47D cell line (Dr. P Dallas, Telethon Kids Institute) were all maintained in a humidified incubator at 37 °C with 5% CO_2_. HEK-293 and A431 cells were cultured in DMEM (Life Technologies) supplemented with 10% heat-inactivated FCS (Rowe Scientific), 2 mM Glutamax (Life Technologies), 100 U/ml penicillin (Life Technologies), and 100 µg/ml streptomycin (Life Technologies). CHO-K1, T47D and AMO-1 cells were cultured in RPMI 1640 (Life Technologies) supplemented with 10% heat-inactivated FCS, 2 mM Glutamax, 100 U/ml penicillin, and 100 µg/ml streptomycin. HL-60 cells were cultured in RPMI 1640 supplemented with 20% heat-inactivated FCS, 100 U/ml penicillin, 100 µg/ml streptomycin, 2 mM Glutamax. Stable cell lines HEK-293/EGFR, HEK-293/BirA, and HEK-293/EGFR-BirA were made by standard transfection and selection techniques, and were cultured in HEK-293 complete medium additionally supplemented with 300 µg/ml Geneticin (Life Technologies), 500 µg/ml Geneticin, or 300 µg/ml Geneticin with 50 µg/ml Hygromycin B (Life Technologies), respectively. CHO-K1/EGFR stable cell line was made using FlpIn™ technology (Thermo Fisher Scientific) and cultured in F-12K medium (Life Technologies) supplemented with 10% heat-inactivated FCS, 100 U/ml penicillin, 100 µg/ml streptomycin, 2 mM Glutamax and 800 µg/ml Hygromycin B. Stable cell line A431/BirA (made by lentiviral infection, Genscript) was cultured in A431 complete medium additionally supplemented with 1 µg/ml Puromycin (Life Technologies). HEK293/GFP1–10 and CHO-K1/GFP1–10 stable cell lines were cultured in complete media for the parental cell line supplemented by 200 µg/ml and 250 µg/ml Zeocin (Life Technologies), respectively. H-2Kb-tsA58 myogenic immortalized cell line (Dr. K Farmer, University of Texas Southwestern Medical Center, and Dr. Terry Partridge, Westminster Medical School) was cultured according to published protocols^59^.

### GFP complementation

GFP complementation assays and fluorescence microscopy were performed as previously described^35^ in HEK-293 or CHO-K1 cells transiently transfected with hGFP1–10 plasmid, or in CHO-K1/GFP1–10 or HEK-293/GFP1–10 stable cell lines. CHO-K1 cells were seeded at 5 × 10^4^ cells/well for microscopy and treated with 10 µM recombinant protein. Images were collected through 100X or 40X DIC objectives.

### Recombinant protein expression and purification

Information and sequences of recombinant proteins used in this study are summarized in Supplementary Tables 4 and 5. Cargo recombinant proteins (Supplementary Table 4) were expressed as His_6_-N-terminally tagged fusions in *E*. *coli* strain BL21 (DE3) Gold (Agilent Technologies), purified using IMAC and filter-sterilized as previously described^35^. An additional purification step was performed for some proteins after IMAC using Ion Exchange Chromatography (IEX). Proteins with an isoelectric point (pI) above 8 were desalted into binding buffer containing 20 mM Na Phosphate, 120 mM NaCl, 10% glycerol (pH 8.0) and loaded on a HiTrap SP HP 5 mL column (GE Healthcare). Proteins with lower pIs were desalted into 20 mM Tris, 50 mM NaCl, 10% glycerol, pH 8.0 binding buffer and purified through a HiTrap Q HP 5 ml column (GE Healthcare). All proteins were eluted using a 0–1 M NaCl gradient. Final proteins were desalted into PBS pH 7.4 and stored in Protein LoBind Eppendorf tubes (Eppendorf, #022431102) as undiluted stocks at −20 °C (short term) or at −80 °C (long term). Protein purity was confirmed by analysis on 4–16% SDS-PAGE stained with Gel Code Blue Stain (Thermo Fisher Scientific).

Recombinant Omomyc proteins were expressed and purified similarly by the UQ Protein Expression Facility (University of Queensland, Australia). Omomyc proteins were expressed with an N-terminal TRX-6XHis tag that was cleaved using HRV3C (Human Rhinovirus 3C protease) protease. After IMAC purification, fractions containing the protein of interest were pooled and desalted into IEX buffer. Tag cleavage was performed overnight at 4 °C with low agitation. Cleaved Omomyc proteins were further purified using IEX, desalted into PBS (pH 7.4), and analyzed as described above.

PAS_SpyC protein was produced in *E*. *coli* KS272^60^ using an 8 L benchtop fermenter with a glucose minimal medium according to a published procedure^51^. In brief, the fermenter was inoculated and bacteria were cultivated at 25 °C with 100 mg/L ampicillin. At OD_550_ = 40 gene expression was induced with 0.5 mM isopropyl β-D-1-thiogalactopyranoside (IPTG) and 1 g/L L-proline was added, followed by cultivation for 2.5 h. After cell harvest by centrifugation, the sediment (wet weight 880 g) was resuspended in 2 L phosphate-buffered saline (PBS; 4 mM KH_2_PO_4_, 16 mM Na_2_HPO_4_, 115 mM NaCl, pH 7.4) with 5 mM dithiothreitol (DTT) and 25 mM EDTA. Then, cell disruption was performed using a PandaPLUS 2000 homogenizer (GEA Niro Soavi), and the cellular debris was separated by centrifugation (SLA 3000 rotor; 11,000 RPM; 4 h; 4 °C). The PAS_SpyC protein was precipitated with 1.2 M ammonium sulfate and dissolved in 1 L PBS. After dialysis against 15 L 20 mM MES/NaOH (pH 5.5), 1 mM EDTA, the protein was subjected to a subtractive anion exchange chromatography using a 500 ml Fractogel EMD SO_3_^-^ column (Merck Millipore). After dialysis against 20 mM Tris/HCl pH 8.0, the protein was purified on a 60 ml MacroCap Q anion exchange column (GE Healthcare), yielding 978 mg at >90% purity. The molecular diameter of PAS_SpyC was examined by applying the protein under reducing conditions to analytical size-exclusion chromatography (SEC) and via dynamic light scattering (DLS) measurements as previously described^61^. For maleimide conjugation of the free Cys residue at the N-terminus, PAS_SpyC was reduced using 2 mM DTT and dialysed against 20 mM sodium phosphate (pH 5.5), 150 mM NaCl, 100 µM EDTA. Aminoethyl-maleimide was dissolved in dimethylformamide and added in a 7-fold molar excess to the protein solution. The coupling reaction was started by adding 12% (v/v) 0.5 M Na_2_HPO_4_, resulting in the appropriate pH of 7.2 for the coupling reaction, which lasted for 48 h. The conjugated protein was dialysed against 20 mM Tris/HCl (pH 8.0) and purified using a 60 ml Source 15 Q AIX column. Final protein concentrations were determined by bicinchoninic acid (BCA) assay (Pierce™).

### Peptides, synthesis and SpyCatcher conjugation

Synthetic peptides were produced by Mimotopes (Supplementary Tables 6,7) and Pepscan (Supplementary Table 8). Peptide-PMOs were produced by Cambridge Research Biochemicals. With the exception of PAS_SpyC proteins, SpyCatcher conjugations were performed with a SpyC protein: SpyT peptide ratio of 1: 1.25, at a 40 µM final concentration for the SpyC protein or 50 µM final concentration for SpyC_PAP conjugations. Conjugation reactions were incubated for 2 h at 22 °C with gentle mixing and then left overnight at 4 °C. Conjugation efficiencies were analyzed on 4–16% SDS-PAGE gels stained with Gel Code Blue Reagent (Thermo Fisher Scientific). PAS_SpyC proteins were conjugated with SpyTag peptides at a ratio of 1: 1.1, and incubated at room temperature for 30 min before overnight incubation at 4 °C.

### Cell viability assays

All cell viability studies were performed in the presence of FCS. Cells were seeded at 2.5–5 × 10^3^ cells/well, depending on cell line, in 96-well plates (PAP assays: CHO-K1 at 3 × 10^3^ cells/well; peptide cytotoxicity assays: CHO-K1 at 5 × 10^3^ cells/well; Bouganin assay: CHO-K1 and CHO-K1/EGFR at 2.5 × 10^3^ cells/well; ^D^PMIα assays: T47D at 5 × 10^3^ cells/well; Omomyc assays: all cell lines at 5 × 10^3^ cells/well). In brief, adherent cells were allowed to adhere for 24 h prior to the addition of treatments whereas suspension cell lines were treated immediately following seeding. Following 2–48 h incubations with treatments, cell viability was measure by a variety of methods. Membrane integrity was assessed by the release of LDH into the media via the CytoTox-ONE reagent (Promega). Metabolic activity was measured either by resazurin reduction potential using PrestoBlue (Life Technologies) or by ATP activity using CellTitre-Glo (Promega). All assays followed manufacturer’s instructions. IC_50_ values were calculated using Prism (version 7.0a, GraphPad).

### Serum parameters of kidney and liver toxicity testing

All mouse studies were approved by the Animal Ethics Committee of The University of Western Australia in accordance with the *Australian Code of Practice for the Care and Use of Animals for Scientific Purposes* (NH&MRC, 8^th^ Edition, 2013). Studies were performed using adult male C57BL/6J mice weighing approximately 20 g. Mice were treated with 1746c27 (40 mg/kg/day intraperitoneally) for 7 days (day 1–7), followed by no treatment on days 8–15. On day 15, mice were anesthetized with intraperitoneal injection of pentobarbitone sodium (240 mg/kg), and their terminal blood collected. Serum was extracted and used to measure kidney and liver toxicity. Urea and creatinine concentration was assessed using QuantiChrom Urea and Creatinine assay kits, respectively (BioAssay Systems). Aspartate transaminase (AST) and alanine transaminase (ALT) concentration were measured using EnzyChrom Aspartate Transaminase and Alanine Transaminase assay kits, respectively (BioAssay Systems). All assays were performed as per manufacturer’s instructions, using a spectrophotometer (PowerWave XS).

### β-lactamase bioassay

β-lactamase internalization assays were performed as previously described^40^. Briefly, SpyC_BLA was conjugated to 1746c27_SpyTag peptide as described above (conjugation efficiencies ≥90%). For flow cytometry assays, CHO-K1 cells (seeded at 1 × 10^5^ cells/well in 24-well plates) in the presence of sera were incubated with SpyC_BLA or SpyC_BLA/CPP_SpyT conjugates at 37 °C/5% CO_2_ for 2 hours. Cells were washed, detached by 5 min incubation with trypsin, washed, loaded with the β-lactamase substrate CCF2-AM (Thermo Fisher Scientific) and analysed by flow cytometry (BD Fortessa); intracellular β-lactamase activity caused an emission shift from 510 nm to 450 nm. The percentages of β-lactamase positive cells for each sample were graphed against the concentration of protein added to the cells.

### PMO exon-skipping assay

Exon skipping assays and RT-PCR detection were performed according to published protocols^59^, transfecting differentiated murine *H-2K*^b^*-tsA58* myogenic cultures with 25 nM to 1 µM of 1746c27_M23D(+7–18) or M23D(+7–18) morpholino^53^ alone. Transfected cells were subsequently incubated in sera-free medium for 4 days before RNA was extracted.

### Systemic delivery of PMOs

Animal experiments and the detection of dystrophin expression by fluorescence microscopy were carried out according to published protocols^62^. Mice (initially 3–5 days of age) were treated with five intra-peritoneal injections of 1746c27_M23D(+7–18) or M23D(+7–18) morpholino, at 4 nmol per dose over two weeks. Each treatment group consisted of two animals (n = 2). The injected volume increased with the increased weight of the mice over the two-week period of the five injections, such that 2 microliters per gram were used. Two weeks after cessation of treatment, tissue samples were taken for detection of dystrophin by fluorescence with a dystrophin polyclonal antibody (Abcam, #ab15277).

C57BL/10ScSnArc^*mdx*^ mice carry a nonsense mutation in exon 23 of the *dystrophin* gene. Control wild type mice are C57BL10/ScSnArc. All mice were supplied by the Animal Resources Centre (Murdoch, Western Australia) and housed according to National Health and Medical Research Council (Australia) guidelines. All animal work was approved and carried out under Murdoch University Animal ethics permit number R2625/13.

### Statistical information

Where appropriate, comparisons and statistical significance were calculated using Prism (version 7.0a, GraphPad). Statistical tests and results (e.g., *P* values) are described in figure legends and use confidence intervals of 95%.

## Electronic supplementary material

Supplementary Figures

Supplementary Tables

## Data Availability

The authors declare that the data supporting the findings of this study are available within the paper and its supplementary information files.
