# Supplementary material for: A platform for discovery of functional cell-penetrating peptides for efficient multi-cargo intracellular delivery
Source: Sci Rep. 2018 Aug 22;8:12538. doi: 10.1038/s41598-018-30790-2 (PMC6105642; doi:10.1038/s41598-018-30790-2)
Supplement: Supplementary file 1 — Supplementary Figures [file 41598_2018_30790_MOESM1_ESM.docx]

# **Supplementary Figures**

# A platform for discovery of functional cell-penetrating peptides for efficient multi-cargo intracellular delivery

Katrin Hoffmann^#^, Nadia Milech^#,*^, Suzy M Juraja^#^, Paula T Cunningham^#^, Shane R Stone, Richard W Francis, Mark Anastasas, Clinton M Hall, Tatjana Heinrich, Heique M Bogdawa, Scott Winslow, Marie N Scobie, Robert E Dewhurst, Laura Florez, Ferrer Ong, Maria Kerfoot, Danie Champain, Abbie M Adams, Susan Fletcher, Helena M Viola, Livia C Hool, Theresa Connor, Brooke AC Longville, Yew-Foon Tan, Karen Kroeger, Volker Morath, Gregory A Weiss, Arne Skerra, Richard M Hopkins and Paul M Watt.

*# Shared first authorship; * Corresponding author*

[Supplementary Figures 1](#_Toc513822554)

[Supplementary Fig. 1: Validation of the CPP discovery platform components. 2](#_Toc513822555)

[Supplementary Fig. 2: Validating expression of BirA by HEK-293/BirA stable cell line. 5](#_Toc513822556)

[Supplementary Fig. 3: EGFR expression in cell lines. 7](#_Toc513822557)

[Supplementary Fig. 4: Principal Component Analysis (PCA) of Phylomer CPP properties. 8](#_Toc513822558)

[Supplementary Fig. 5: Biophysical characteristics of Phylomer CPPs. 10](#_Toc513822559)

[Supplementary Fig. 6: Circular dichroism spectra of Phylomer CPPs. 11](#_Toc513822560)

[Supplementary Fig. 7: Confocal live cell imaging visualizes 1746c27-mediated uptake of β-lactamase 12](#_Toc513822561)

[Supplementary Fig. 8: 1746c27-delivered Omomyc is more potent than MYC small molecule inhibitors. 13](#_Toc513822562)

[Supplementary Fig. 9: Evidence of exon skipping for dystrophin *in vivo* by RT-PCR and Western blot. 14](#_Toc513822563)

[Supplementary Methods 15](#_Toc513822564)

[Cell culture and fluorescence microscopy 15](#_Toc513822565)

[Flow cytometry 15](#_Toc513822566)

[Bioinformatics and code availability 15](#_Toc513822567)

[Circular dichroism spectra analysis 15](#_Toc513822568)

[Confocal live-cell microscopy 16](#_Toc513822569)

[PMO exon-skipping assay (in vivo samples) 16](#_Toc513822570)

[Cell fractionation and immunoblotting 16](#_Toc513822571)

[Cell fractionation and immunoblotting: detection of BirA and Rab7 protein 16](#_Toc513822572)

[Immunoblotting: detection of dystrophin protein 16](#_Toc513822573)

[Bibliography (Supplementary Figures) 18](#_Toc513822574)

## Supplementary Fig. 1: Validation of the CPP discovery platform components.

**1a. Phage biotinylation is dependent on display of the Avitag peptide sequence.**

Phage are exposed to endogenous biotinylation by the natural biotin protein ligases in *E. coli* during culture growth.

Aliquots of 1.98x10^9^ pfu of T7 phage lysates were separated by SDS-PAGE through a 10% (w/v) polyacrylamide Bis-Tris gel, transferred to PVDF membrane, and probed with streptavidin-HRP. T7 phage Biotinylation is dependent on display of the Avitag sequence (lanes 1-3); wildtype T7 phage is not biotinylated (lane 4).

*Lane 1:* EBD displayed C-terminal to Phylomer.

*Lane 2:* Avitag sequence displayed.

*Lane 3:* EBD displayed N-terminal to Phylomer.

*Lane 4:* Wildtype T7 (10b capsid).

**1b. *Ex-vivo* biotinylation of T7 Avitag phage spiked into mammalian cell lysate.**

Biotinylation of T7 Avitag phage can occur in lysates from HEK-293/BirA cells and is improved by addition of biotin to cells or lysate (positive controls). This result verifies that the mammalian-expressed BirA enzyme is functional inside cells or cell lysates.

Aliquots of 2x10^6^ cells from HEK-293 or HEK-293/BirA cell lines were incubated with or without 50 µM d-biotin for 1 h at 37 ˚C in 5% CO_2_ prior to lysis. Cells were lysed and clarified. Then 1.1x10^10^ purified T7 Avitag phage were added to cell lysate and incubated at 30 ˚C for 30 min, with or without 50 µM d-biotin. Samples were equally loaded and separated by SDS-PAGE through a 10% (w/v) polyacrylamide Bis-Tris gel, transferred to PVDF membrane, and probed with streptavidin-HRP.

*Lanes 1-2:* Lysate from HEK-293 or HEK-293/BirA cells was mixed with T7 Avitag phage; phage are biotinylated in the lysate of HEK-293/BirA cells

*Lanes 3-4:* Lysate from HEK-293 or HEK-293/BirA cells where cells were pre-incubated with d-biotin before lysis and mixing with T7 Avitag phage; phage are biotinylated in the lysate of HEK-293/BirA cells and the biotinylation signal is improved by pre-incubation with d-biotin

*Lanes 5-6:* Lysate from HEK-293 or HEK-293/BirA cells was mixed with T7 Avitag phage and d-biotin added into the lysate mix; phage are biotinylated in the lysate of HEK-293/BirA cells and the biotinylation signal is improved by addition of d-biotin

**1c. Reduction of endogenous biotinylation in Avitagged T7 libraries by growth in *E. coli* strain containing the pSUMO-Avi3 decoy construct.**

The endogenous biotinylation of Avitagged T7 phage vectors by natural biotin protein ligases in *E. coli* can be reduced by culturing phage growth in cells containing a pSUMO-Avi3 decoy construct. The results show preferential biotinylation of the decoy Sumo-Avi3 protein over Avitagged phage from different N-terminal (N’) display libraries (lanes 1-4) and empty Avitagged T7 phage vector (lane 6) compared to Avitagged T7 phage expressed in cells without the decoy construct (lane 5).

Equal amounts of cleared T7 phage lysates were separated by SDS-PAGE through a 10% (w/v) polyacrylamide Bis-Tris gel, transferred to PVDF membrane, and probed with streptavidin-HRP. Biotin carboxyl carrier protein (BCCP) is the endogenous *E. coli* protein that is biotinylated by BirA biotin ligase^1^. The libraries (lanes 1-4) were constructed using genomic material from either archaea (A) or bacteria (B) microbes.

*Lanes 1-2:* Lysate from T7 libraries T08 and T09 grown in *E.coli* strain 5615 with decoy construct pSUMO_Avi3 (Supplementary Table 2a); libraries display an EBD_HA_Avitag sequence N-terminal to the Phylomer.

*Lanes 3-4:* Matched libraries from lanes 1-2, grown in *E.coli* strain 5615 with decoy construct pSUMO-Avi3, only without the EBD fragment (i.e., libraries display a HA_Avitag sequence N-terminal to the Phylomer).

*Lanes 5-6*: T7 phage lysate of a clone displaying EBD_HA_Avitag and grown in *E.coli* 5615 or in *E.coli* strain 5615 with decoy construct pSUMO_Avi3, respectively.

**1d. Endogenous biotinylation for Avitagged T7 libraries in presence and absence of pSUMO-Avi3 decoy.**

The endogenous biotinylation of Avitagged T7 phage libraries by natural biotin protein ligases in *E. coli* can be reduced by culturing phage growth in cells containing a pSUMO-Avi3 decoy construct. The image shows phage made from empty library vector constructs and matched, exemplar T7 phage libraries, where phage displayed EBD_Avitag either C-terminal (C’) or N-terminal (N’) to the Phylomer, respectively.

Equal amounts of cleared T7 phage lysates grown in the presence or absence of pSUMO-Avi3 decoy construct were separated by SDS-PAGE through a 10% (w/v) polyacrylamide Bis-Tris gel, transferred to PVDF membrane, and probed with streptavidin-HRP. The libraries (lanes 5-9) were constructed using genomic material from bacteria (B).

*Lanes 1:* Cell lysate from *E.coli* strain 5615 with the decoy construct pSUMO_Avi3.

*Lanes 2-5:* Lysates of T7 phage empty vector constructs grown in *E.coli* decoy strain 5615 ± pSUMO_Avi3; T7 phage display the EBD_Avitag fusion protein either C-terminal (C’) or N-terminal (N’) to the cloning sites used in Phylomer library construction.

*Lanes 6-9:* Matched T7 library lysates from lanes 2-5, grown in *E.coli* decoy strain 5615 ± pSUMO_Avi3; T7 phage display the EBD_Avitag fusion protein either C-terminal (C’) or N-terminal (N’) to the Phylomer peptides.

**1e. Inhibition of BirA activity in cell lysate.**

HEK-293/BirA cells were lysed and a 10 µM excess of biotin added. PEG-purified T7 EBD_HA_Avitag phage grown in *E.coli* strain 5615 with decoy construct pSUMO_Avi3 (1x10^9^ pfu) were added to lysed cell preparation (2x10^5^ cells) in the presence of decreasing concentrations of pyrophosphate (PPi) and incubated at 30 ˚C for 30 min. Samples were separated by SDS-PAGE through a 10% (w/v) polyacrylamide Bis-Tris gel, transferred to PVDF membrane, and probed with streptavidin-HRP.

## Supplementary Fig. 2: Validating expression of BirA by HEK-293/BirA stable cell line.

**2a. Functional BirA expression in HEK-293/BirA stable cells.**

Expression of functional BirA in the HEK-293/BirA stable cell line was confirmed by transfecting cells with a plasmid expressing β-actin tagged with an Avitag. Successful biotinylation of the Avitag by the BirA in the cells was detected by staining the cells with FITC-labelled streptavidin. Fluorescence microscopy identified the streptavidin binding to the biotinylated Avitag (FITC-channel). Cells are counter-stained for endogenous β-actin (TRITC) and nuclei (DAPI). Cell images were captured at 100X or 40X magnification.

Cells that stained negative for biotinylated Avitag (FITC negative) show β-actin expression localized to the cytoskeletal architecture (for example, TRITC staining at actin filaments). Cells that stained positive for biotinylated Avitag (FITC positive) show β-actin expression throughout the cell (TRITC staining through the cytoplasm). This illustrates that the positive FITC signal was not due to non-specific staining of the cells.

**2b. BirA is expressed only in the cytoplasm of HEK-293/BirA stable cells.**

Aliquots of 4 µg of cytosolic and membrane soluble cell fractions from HEK-293/BirA and HEK-293 cell lines were separated by SDS-PAGE through a 4-12% (w/v) polyacrylamide Bis-Tris gel, transferred to PVDF membrane, and immunoblotted. BirA (35 kDa) protein expression was only detected in the cytosolic fraction of lysates from stable cell line HEK-293/BirA; no BirA expression was detected in HEK-293 cells. Rab7 (23 kDa) protein expression can be used as a marker for late endosomes and is a key regulator of endosome maturation and late endosome trafficking (see review^2^). Consistent with this, Rab7 expression was detected in the membrane fraction of both HEK-293 and HEK-293/BirA cells but not in the cytoplasmic fraction.

Legend: C = cytosolic fraction (4 µg); M = membrane fraction (4 µg).


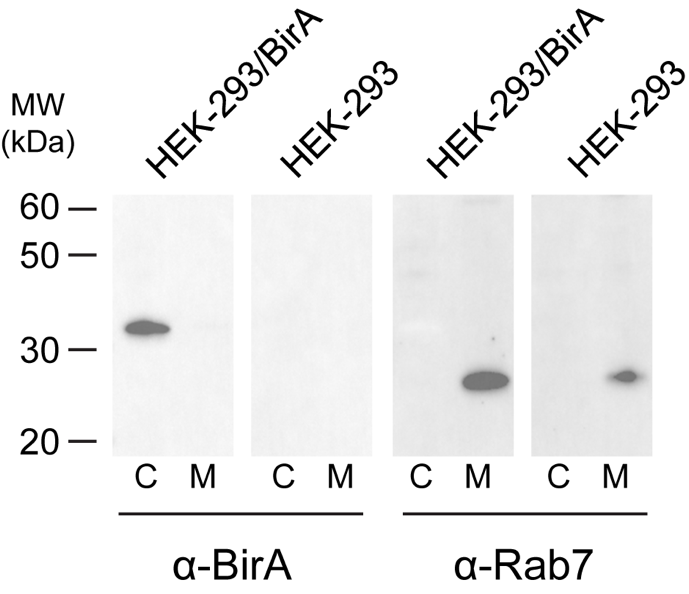


## Supplementary Fig. 3: EGFR expression in cell lines.

EGFR expression in A431, HCC-827, HEK-293/EGFR, HEK-293, CHO-K1, and CHO-K1/EGFR cells was assessed by flow-cytometry using a FITC-labelled antibody that specifically binds to human EGFR (hEGFR). As expected, CHO-K1 cells did not bind to this antibody, and can be considered hEGFR-negative. In contrast, A431 cells showed the largest shift to the right on the FITC axis (X axis) indicative of their relatively high surface expression of hEGFR. The relative surface expression of hEGFR detected in the three other cell lines fell within this range: HEK-293 cells showed low expression; HCC-827 and CHO-K1/EGFR stable cells showed intermediate expression; and HEK-293/EGFR stable cells showed intermediate-to-high hEGFR expression.


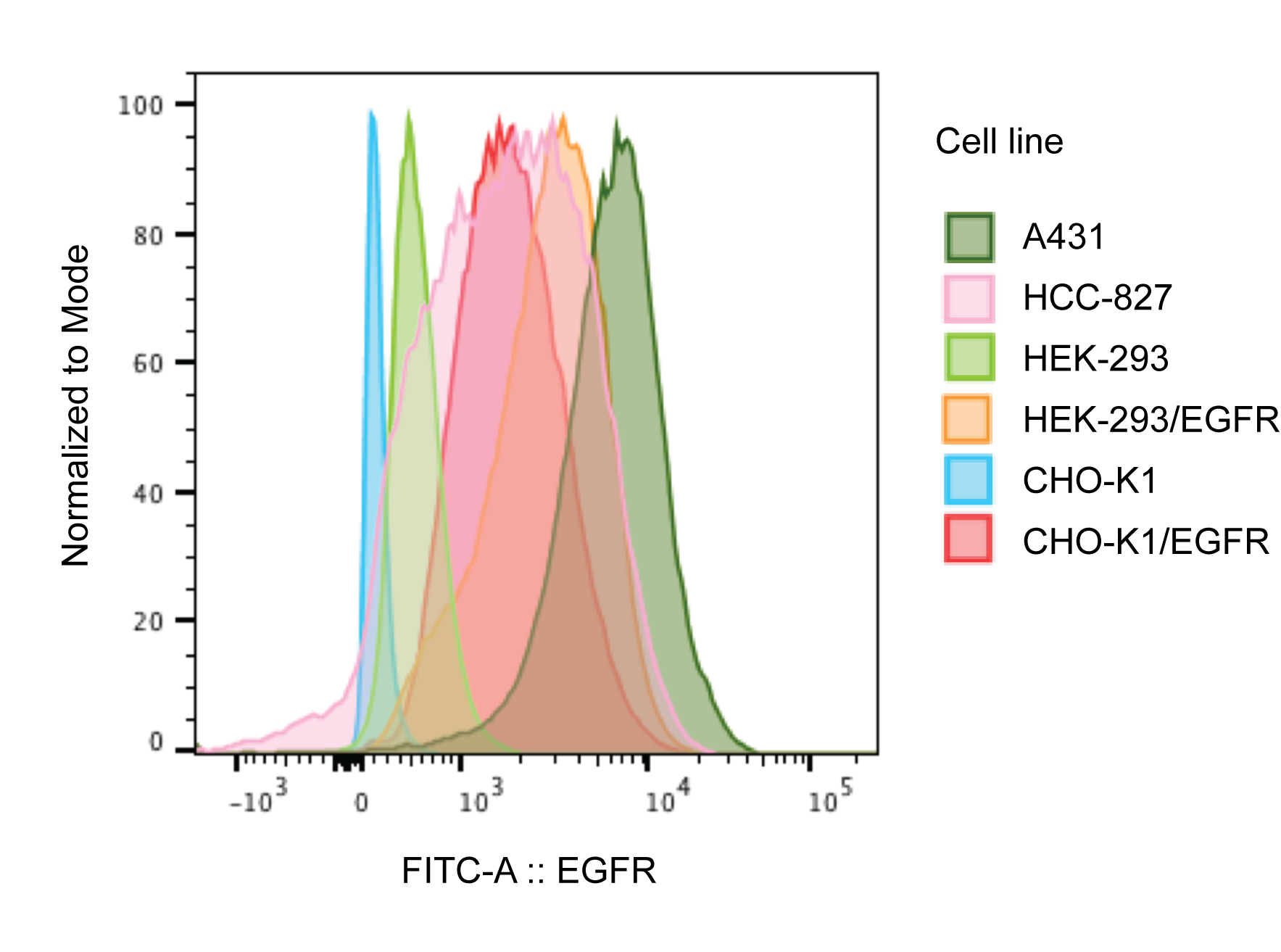


## Supplementary Fig. 4: Principal Component Analysis (PCA) of Phylomer CPP properties.

Principal Component Analysis of a wide range of peptide properties for Phylomer CPP sequences versus those from either (**a**) naïve Phylomer libraries, (**b**) canonical CPPs, or (**c**) both. These analyses show clustering of Phylomer CPPs distinct from canonical CPPs. Canonical CPPs were sourced from the literature^3-6^ and consists of 59 unique primary CPPs; this subset does not include D-peptide, non-proteinogenic or NLS sequences. Naïve library sequences consist of 75 random Phylomer peptides from the library (≥10 aa).


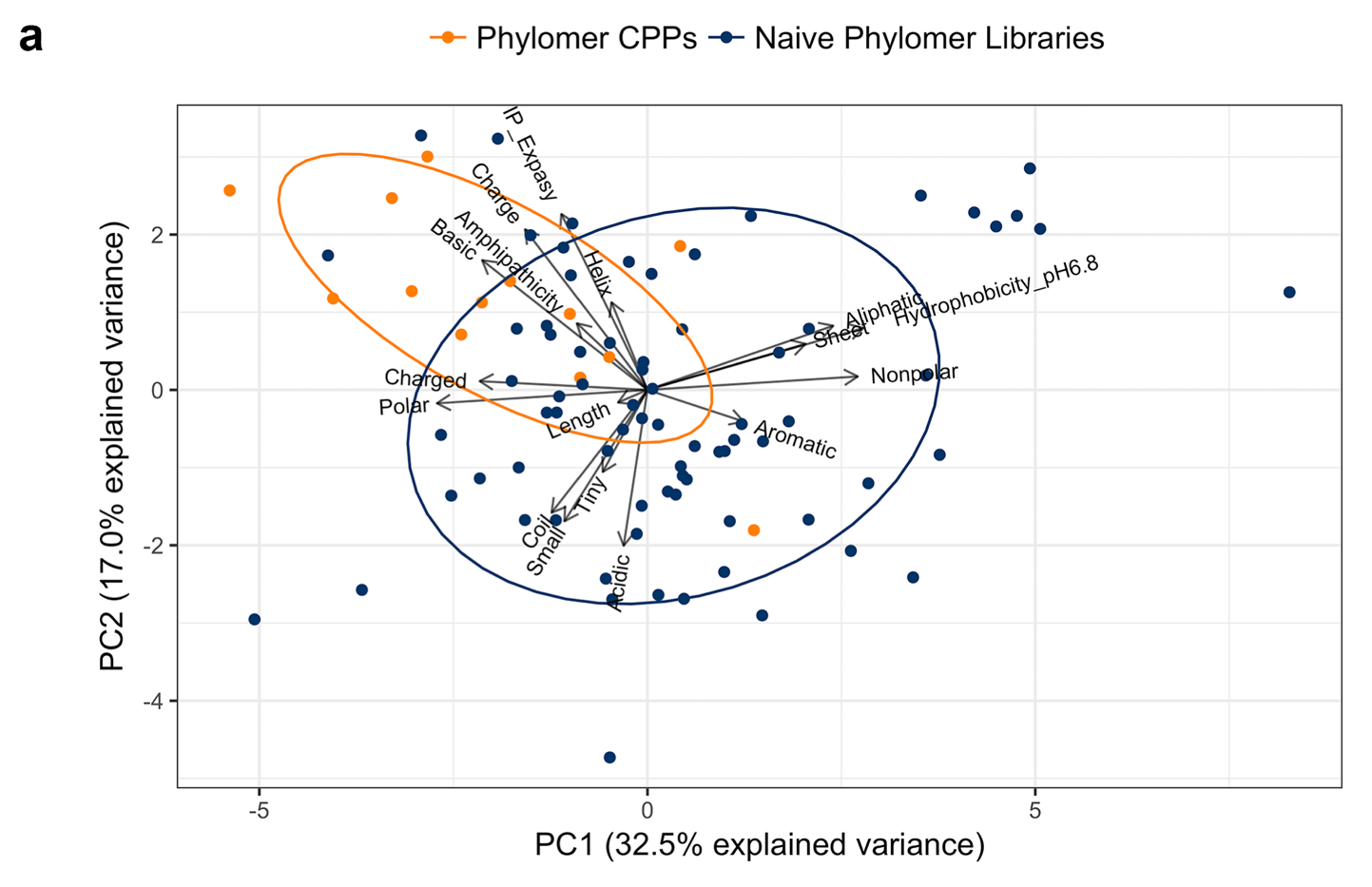


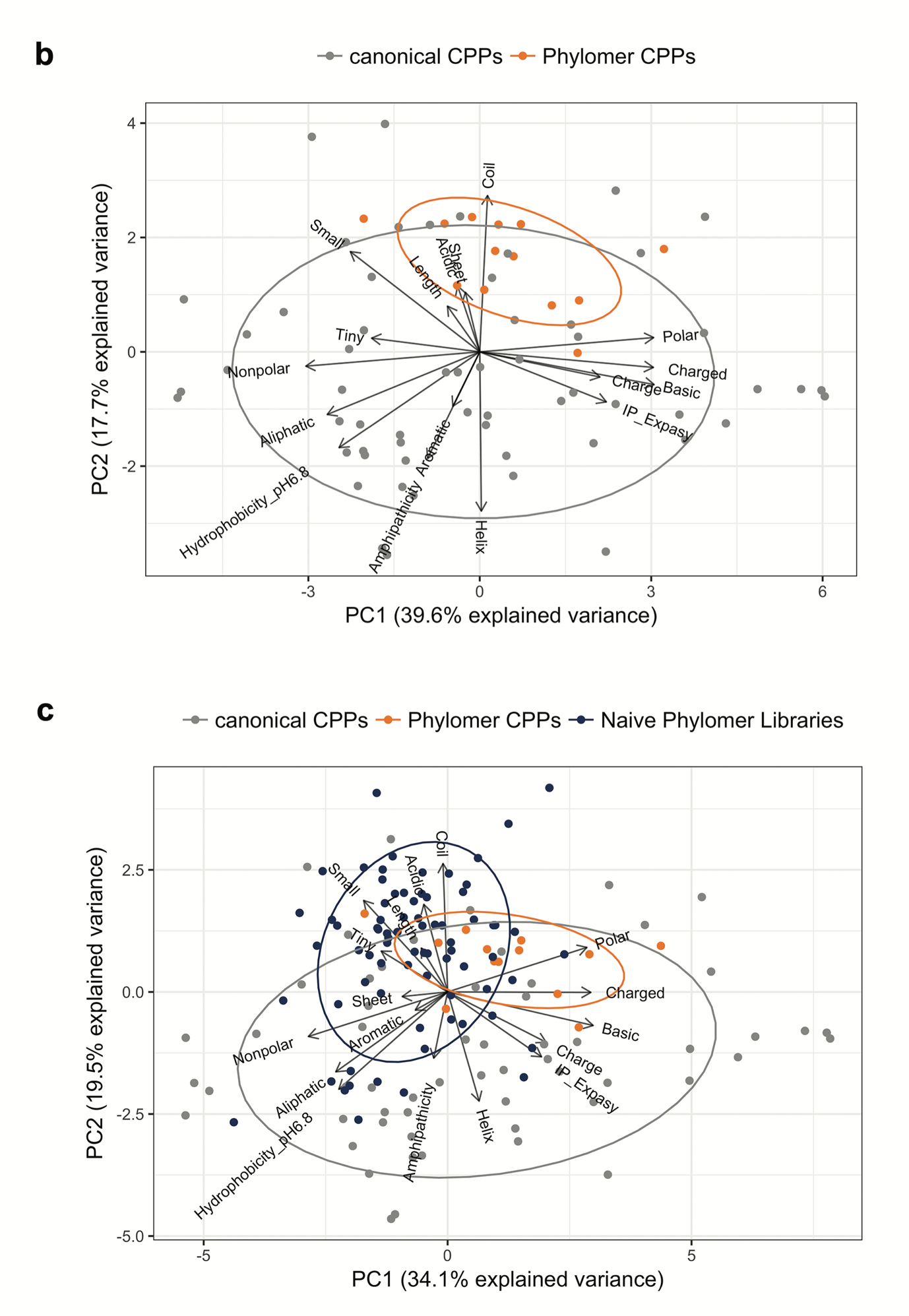


## Supplementary Fig. 5: Biophysical characteristics of Phylomer CPPs.

Specific comparison of biophysical characteristics show that compared to canonical CPPs, Phylomer CPPs have (**a**) increased average length (p < 0.0001), (**b**) higher positive charge (p = 0.0029), and (**c**) lower hydrophobicity (p = 0.01). (**d**) Comparing average amino acid compositions shows an increase in lysine (K, p = 0.021), proline (P, p = 0.006), glutamine (Q, p = 0.006) and serine (S, p = 0.014) residues in Phylomer CPP sequences compared to canonical CPPs. The increase in lysine residues will be an influential factor in the increased positive charge seen in Phylomer CPPs. This statistical analysis applies an unpaired two-tailed t-test.


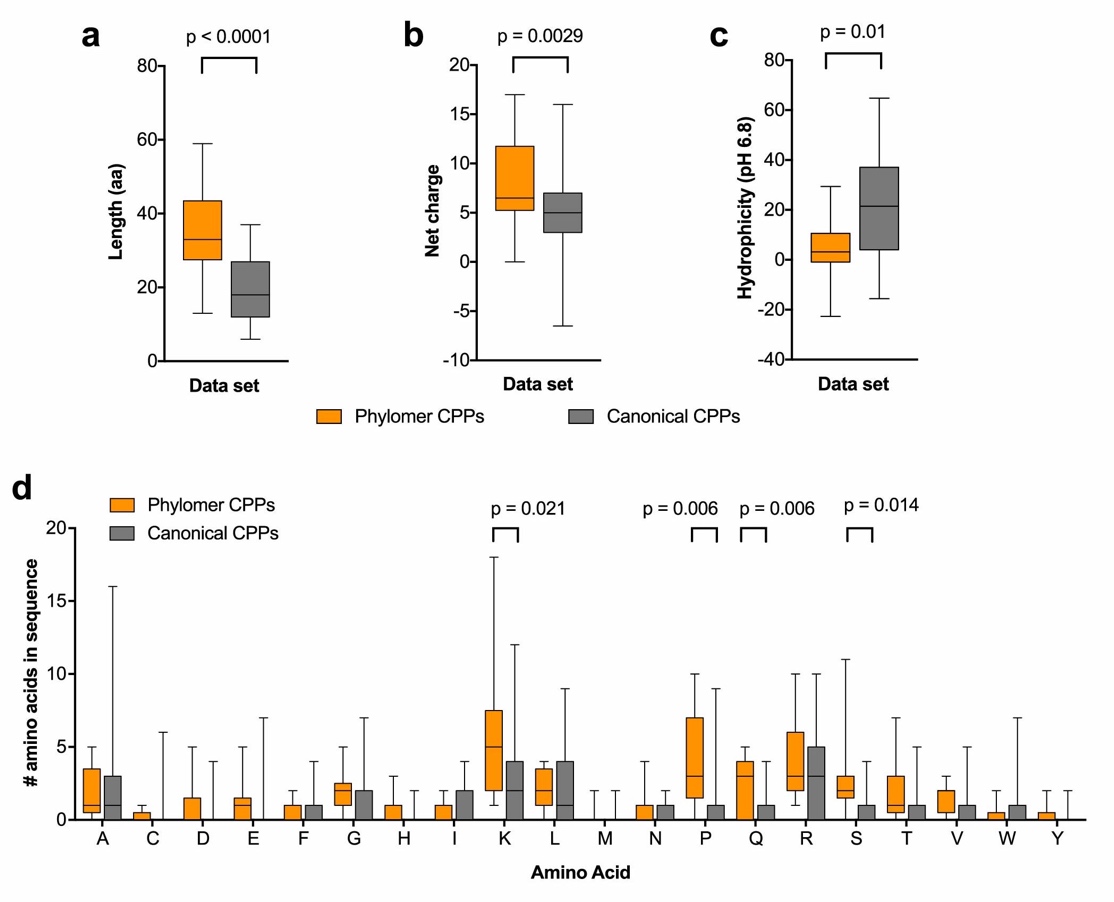


## Supplementary Fig. 6: Circular dichroism spectra of Phylomer CPPs.

Phylomer CPPs are largely random coiled and not highly structured. Circular dichroism spectra of various cell penetrating peptides display unique and varied conformational characteristics at different pH (pH 4.5 and pH 7.2) and in the presence of SDS micelles (pH 7.2, 10 mM SDS). (**a**) ^FITC^1746; (**b**) ^FITC^0084; (**c**) ^FITC^1869; (**d**) ^FITC^TAT, (**e**) 1746; and (**f**) 1746c27. All experiments were conducted at 25 ˚C.

**a:** ^FITC^1746 is predominantly coil at pH 4.5, pH 7.2, and in the presence of SDS micelles.

**b:** ^FITC^0084 displays a combination of coil and helix secondary structure at pH 7.2, which shifts to increased helix in the presence of SDS micelles. At pH 4.5, the structure shifts strongly to predominantly coil.

**c:** ^FITC^1869 at pH 7.2, displays a combination coil and turn structure. In the presence of SDS micelles, the structure shifts to increased helicity. At pH 4.5, the structure shifts to being predominantly coil.

**d:** ^FITC^TAT displays coil like secondary structure at pH 7.2 and in the presence of SDS, but undergoes a shift at pH 4.5 unrelated to known secondary structure.

**e:** 1746 spectra is comparable to ^FITC^1746, and the peptide structure is predominantly coil at all conditions; data for pH 4.5 (orange) and pH 7.2 (red) overlap.

**f:** 1746c27 spectra is comparable to 1746, and the structure of the truncated variant remains predominantly coil at all conditions; data for pH 4.5 (orange) and pH 7.2 (red) overlap.

## Supplementary Fig. 7: Confocal live cell imaging visualizes 1746c27-mediated uptake of β-lactamase

T47D cells were treated with either 1746c27-conjugated or unconjugated SpyC_β-lactamase enzyme at concentrations of (**a, b**) 2 µM, (**c, d**) 4 µM, or (**e, f**) 8 µM, in the presence of the fluorescent β-lactamase substrate. Substrate cleavage occurs only in cells where beta-lactamase in internalised, visualised as a change from green to blue fluorescence. Using confocal live cell imaging, 1746c27-mediated uptake of SpyC_β-lactamase was dose-dependent and detected at all concentrations of conjugate treatment through observation of substrate cleavage. Live cell uptake of unconjugated SpyC_β-lactamase and subsequent substrate cleavage was not observed. Bar scale is 50 µm.


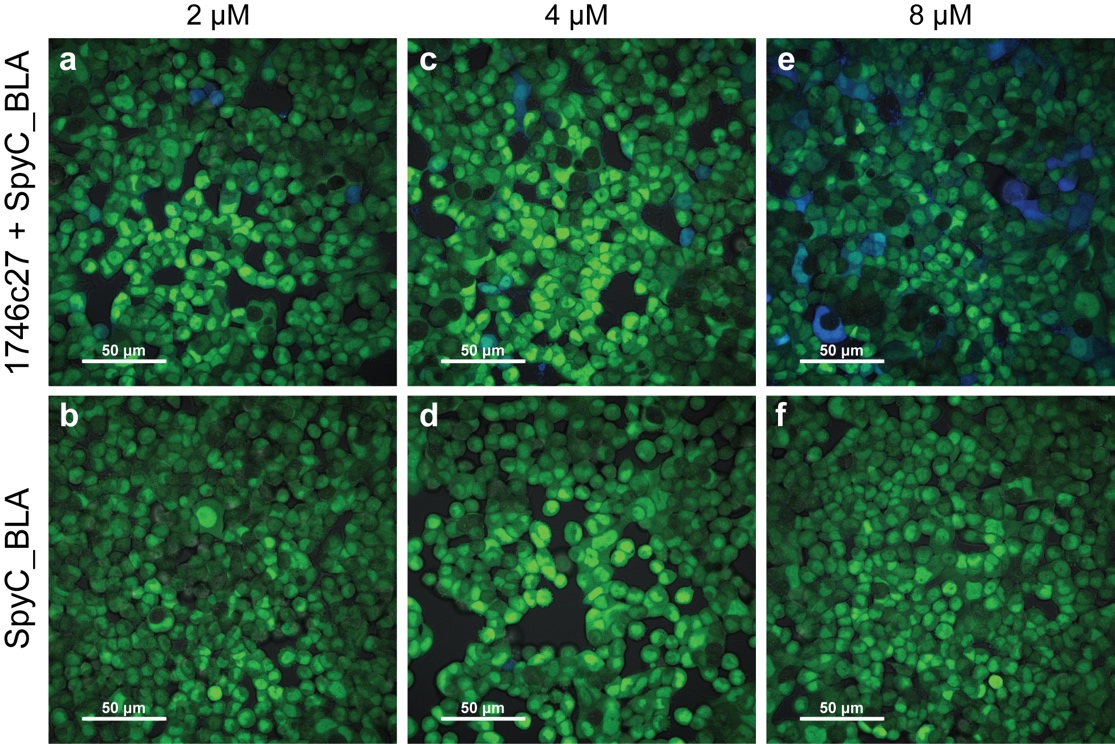


## Supplementary Fig. 8: 1746c27-delivered Omomyc is more potent than MYC small molecule inhibitors.

Treatment of AMO-1 (plasmacytoma), HL-60 (promyelocytic leukemia), and T47D (breast cancer) cell lines with 1746c27_Omomyc and small molecule inhibitors. After 48 h incubation of the compounds on cells, the cell viability was assessed by measuring ATP activity. Results show strong, similar efficacy of 1746c27_Omomyc across all three cell lines that is greater than the potency of MYC small molecule inhibitors 10058-F4^7^ and KJ-Pyr9^8^.

Phylomer 1746c27 alone and DMSO alone (vehicle control for small molecule inhibitors) show no significant cytotoxicity. Control protein Omomyc exhibits a minor effect on cell viability only at the highest concentrations (mid to high micromolar potencies).

Results are from 1 independent experiment. Error bars represent standard deviation from the mean of duplicate samples.

## Supplementary Fig. 9: Evidence of exon skipping for dystrophin *in vivo* by RT-PCR and Western blot.

Exon skipping *in vivo* was evaluated at the **(a, c)** RNA and **(b)** protein level in the Duchenne muscular dystrophy mouse disease model. RNA was extracted from tissues of C57BL/10ScSn*^mdx^* mice treated with five intra-peritoneal injections of CPP_PMO cargo over two weeks at 4 nmoles per dose (injected volume increased with increased weight of the mice). Pip6, a traditional carrier CPP for peptide-oligonucleotide conjugates in the *mdx* disease model of DMD^9^, was tested *in vivo* in parallel for comparison. All samples were loaded onto the gel using normalization by myosin densitometry, to enable a quantitative comparison.^10^

**(a, b)** Exon skipping is only detected 1746c27_M23D(+7-18)_PMO (n=2) treated mice, or those treated with the assay positive control CPP_PMO cargo, Pip6_ M23D(+7-18)_PMO (n=1). In untreated C57BL/10ScSn*^mdx^* mice or those treated with M23D(+7-18) alone, exon 23 skipping was not detected. Consistent with muscle staining for dystrophin expression in tissue sections (Fig. 7b), the strongest dystrophin signal (427 kDa) was detected in RNA extracted from the diaphragm, followed by samples from the *tibialis anterior* and finally heart tissue. **(c)** Exon skipping was not detected in any tissue from mice treated with CPP 1746c27 or Pip6 alone (n=1), measured by RT-PCR.

# Supplementary Methods

## Cell culture and fluorescence microscopy

HEK-293/BirA cells were electro-transfected with plasmid DNA (pcDNA4/TO_β-actin, pcDNA4/TO_β-actin_Avitag) using the Neon transfection system (Life Technologies). Briefly 3x10^5^ cells / 100 µl were prepared according to manufacturer’s instructions and transfected with 0.5 µg DNA with the following pulse conditions: 1100 V, 20ms, 2 pulses. Following transfection, the cells were transferred to 8 well glass chamber slides (Nalge Nunc International) pre-coated with gelatin and were maintained in media +/- 5 µM biotin for 18 h. Media was changed to biotin-free media for 2 h prior to processing to reduce non-specific background. At 20 h post transfection the cells were fixed (4% formaldehyde/PBS) and permeabilized (0.4% Saponin/PBS) for 1 h at 4 ˚C. Successful biotinylation of the Avitag was detected by incubation with Streptavidin-FITC (1:100 in the permeabilization buffer) for 60 min. For wells that were counterstained, a second incubation with β-actin Red 555 Ready Probes Reagent (Life Technologies, #R37112) was performed following manufacturer’s instructions. Wells were then washed and mounted with Vectashield antifade mounting media with DAPI (Vector Laboratories). Images were collected through 100X or 40X DIC objectives using an Olympus BX53 with DP72 camera. Image overlays were compiled using the ImageJ software.

## Flow cytometry

Cells were detached from plates with 0.25% trypsin/EDTA (Gibco), then washed and stained with EGFR-FITC mAb (1:100) (Rat mAb against EGFR with FITC label; Abcam Cat# ab11400, Lot#GR82441-2) for 30 min @ 4 ˚C. Post-staining cells were washed in buffer (PBS/1% FCS) and resuspended in PBS/5% formaldehyde. Cells and data assessed by flow cytometry on the LSRFortessa with FACS DIVA software (BD Biosciences), excited with a 488 nm blue laser, and detected with a 530/30 filter. Data were analyzed with FlowJo X 10.0.7 software (Tree Star Inc.).

## Bioinformatics and code availability

Sanger-sequenced Phylomers were analyzed through proprietary pipelines to obtain translated peptide sequences, associated peptide properties and identify nORFs. Peptide sequences (≥6 aa) were clustered with CD-HIT^11^ at 70% identity and overlapping clusters were identified and merged using a Dynamic Programming approach employing the "ends-free” Miller Myers alignment algorithm^12^. Complete cluster member sequences were aligned using Clustal Omega^13^ and sequence siblings (identical sequences in both length and identity) and overlaps (≥ 10 amino acids and with ≥90% identity, also includes identical sequences) were identified. Statistical comparisons of peptide properties between datasets used a two-sample two-tailed t-test to assess the propensity for certain amino acid compositions depending on the screen attributes. Additional comparisons of peptide properties between combinations of Phylomer CPP sequences, naïve sequences and canonical CPP sequences sourced from the literature were made using Principal Component Analysis using R software (version 3.4.0, The R Foundation).

## Circular dichroism spectra analysis

All peptides were resuspended at 40 µM in 10 mM NaF buffer at pH 4.5 or pH 7.2. For solutions containing SDS, FITC-labelled peptides were at 40 µM in 10 mM NaF, 10 mM SDS, pH 7.2; non-FITC peptides were at 40 µM in 10 mM NaF, 30 mM SDS, pH 7.2. Circular dichroism spectra for FITC-labelled peptides were collected on an OLIS DSM 1000 Circular Dichroism spectrophotometer (Olis, Inc.) equipped with a 6-cell changer and peltier thermal unit. These spectra were collected at 25 ˚C in continuous scanning mode at 1 nm/sec, 1 nm bandwidth, in 1 mm pathlength cuvettes (Starna Pty Ltd). Presented spectra are the average of 10 repeat scans. Circular dichroism spectra for non-labelled peptides were collected on a Jasco-1500 Circular Dichroism spectrophotometer (Jasco, Inc.) at 25 ˚C in continuous scanning mode at 100 nm/min, 0.1 nm bandwidth, in 1 mm pathlength cuvettes (Starna Pty Ltd). Presented spectra are the average of 3 repeat scans.

## Confocal live-cell microscopy

SpyC_BLA was conjugated to 1746c27_SpyTag peptide as described in the Methods (conjugation efficiencies ≥90%). For confocal live cell imaging, T47D cells were seeded at 2x10^5^ cells/ml onto 4-well ibiTreat chamber slides (Thermo Fisher Scientific) and incubated for 48 h at 37 ˚C, 5% CO_2_. Then the media in chamber slides was removed and replaced with 1746c27-conjugated or unconjugated SpyC_BLA protein diluted in complete media (RPMI supplemented with 10% heat-inactivated FCS, 2 mM Glutamax (Life Technologies), 100 U/ml penicillin, and 100 µg/ml streptomycin). Treated cells were incubated for 2 h at 37 ˚C, 5% CO_2_ before the protein-containing media was removed and cells were washed. CCF2-AM substrate was added to cells and incubated for 1 h. Live cells were then visualized through confocal microscopy. Images were collected using a Nikon C2 Confocal Microscope through a 40X DIC objective after excitation by a 408 nm laser. Bar scales in images represent 50 µm.

## PMO exon-skipping assay (in vivo samples)

Tissues were sectioned (6 µm thickness) before adding lysis buffer. Otherwise exon skipping assays and RT-PCR detection were performed following the same protocols used for detection of exon skipping *in vitro* in myoblasts cells (see Methods of main text).

## Cell fractionation and immunoblotting

### Cell fractionation and immunoblotting: detection of BirA and Rab7 protein

Cytosolic and membrane soluble cell fractions from HEK-293/BirA and HEK-293 cell lines were prepared following published protocols^14^ modified with increased digitonin (100 µg/ml) in Lysis buffer A for complete cytosol extraction^15^. Protein concentration of extracted fractions were quantitated (Pierce BCA Protein Assay Kit, Thermo Fisher Scientific) and 4 µg of cytosolic or soluble membrane fractions separated through a 4-12% Bis-Tris gel (Life Technologies). BirA and Rab7 expression in the fractions was detected using monoclonal anti-BirA antibody (Novus Biologicals, cat #NBP2-59938, lot #CRT/17/87) and polyclonal anti-Rab7 antibody (Sigma Aldrich, cat #R4779-200uL, lot #016M4839V), followed by anti-mouse (Life Technologies, cat #31430, lot #SF252846) and anti-rabbit (Life Technologies, cat #A27036, lot #SE247011A). Primary antibodies were diluted at 1 : 5000, while secondary antibodies were diluted to 1 : 100000 (anti-mouse) and 1: 20000 (anti-rabbit). This was followed by detection using SuperSignal West maximum sensitivity substrate (Thermo Fisher Scientific) according to the manufacturer’s instructions. Images were captured on a ChemiDoc Gel Imaging System (BioRad).

### Immunoblotting: detection of dystrophin protein

Samples were suspended at a ratio of 4.5 mg/100 µl of treatment buffer (125 mM Tris/HCl pH 6.8, 15% SDS, 10% glycerol, 50 mM DTT, Bromophenol blue 40 µg/ml, 0.5 mM PMSF and Sigma P8340 protease inhibitors 3 µl/100 µl buffer). Samples were vortexed for 10 sec, sonicated at a setting of 30/100 (Sonics sonicator) for 6 x 1 sec pulses, heated at 95 ˚C for 5 min and centrifuged at 14,000rpm on a Beckman microfuge for 4 min before loading. Total protein was quantitated by BCA assay (Pierce). Average total protein concentration was 2.3 µg/µl. Myosin heavy chain densitometry^10^ was performed on samples that were first loaded on BIS-Tris 4-12% gradient gels. Coomassie stained bands were analysed using Bio1D software and the results were used as a loading control to provide equal loading for the western gel that followed. Average lane loading was 40 µl (92 µg) per lane.

For immunoblotting, proteins were separated through 3-10% Tris-HCl polyacrylamide gels and transferred onto Pall Fluorotrans W membranes (290 mA overnight at 18 ˚C). Dystrophin protein was detected using monoclonal anti-dystrophin antibody (Leica Biosystems, cat #NCL-DYS2, lot #6036626) diluted at 1 : 100 for 2 h at room temperature. This was followed by detection using a Western Breeze kit (Life Technologies) according to the manufacturer’s instructions. Image capture was by Vilber Lourmat Fusion FX gel documentation system followed by analysis of bands using Bio1D software.

# Bibliography (Supplementary Figures)

1. Choi-Rhee, E. & Cronan, J. E. The biotin carboxylase-biotin carboxyl carrier protein complex of Escherichia coli acetyl-CoA carboxylase. *J Biol Chem* **278,** 30806–30812 (2003).

2. Hyttinen, J. M. T., Niittykoski, M., Salminen, A. & Kaarniranta, K. Maturation of autophagosomes and endosomes: a key role for Rab7. *Biochim Biophys Acta* **1833,** 503–510 (2013).

3. Fischer, R., Fotin-Mleczek, M., Hufnagel, H. & Brock, R. Break on through to the other side-biophysics and cell biology shed light on cell-penetrating peptides. *Chembiochem* **6,** 2126–2142 (2005).

4. Heitz, F., Morris, M. C. & Divita, G. Twenty years of cell-penetrating peptides: from molecular mechanisms to therapeutics. *Br J Pharmacol* **157,** 195–206 (2009).

5. Jones, A. T. & Sayers, E. J. Cell entry of cell penetrating peptides: tales of tails wagging dogs. *J Control Release* **161,** 582–591 (2012).

6. Lindgren, M. & Langel, U. Classes and prediction of cell-penetrating peptides. *Methods Mol Biol* **683,** 3–19 (2011).

7. Huang, M.-J., Cheng, Y.-C., Liu, C.-R., Lin, S. & Liu, H. E. A small-molecule c-Myc inhibitor, 10058-F4, induces cell-cycle arrest, apoptosis, and myeloid differentiation of human acute myeloid leukemia. *Exp Hematol* **34,** 1480–1489 (2006).

8. Hart, J. R. *et al.* Inhibitor of MYC identified in a Kröhnke pyridine library. *P Natl Acad Sci USA* **111,** 12556–12561 (2014).

9. Betts, C. *et al.* Pip6-PMO, a new generation of peptide-oligonucleotide conjugates with improved cardiac exon skipping activity for DMD treatment. *Mol Ther Nucleic Acids* **1,** e38 (2012).

10. Nicholson, L. V. *et al.* Dystrophin or a ‘related protein’ in Duchenne muscular dystrophy? *Acta Neurol. Scand.* **86,** 8–14 (1992).

11. Fu, L., Niu, B., Zhu, Z., Wu, S. & Li, W. CD-HIT: accelerated for clustering the next-generation sequencing data. *Bioinformatics* **28,** 3150–3152 (2012).

12. Myers, E. W. & Miller, W. Optimal alignments in linear space. *Comput Appl Biosci* **4,** 11–17 (1988).

13. Sievers, F. *et al.* Fast, scalable generation of high-quality protein multiple sequence alignments using Clustal Omega. *Mol Syst Biol* **7,** 539–539 (2011).

14. Baghirova, S., Hughes, B. G., Hendzel, M. J. & Schulz, R. Sequential fractionation and isolation of subcellular proteins from tissue or cultured cells. *MethodsX* **2,** 440–445 (2015).

15. Holden, P. & Horton, W. A. Crude subcellular fractionation of cultured mammalian cell lines. *BMC Research Notes* **2,** 243 (2009).
