# Supplementary material for: A platform for discovery of functional cell-penetrating peptides for efficient multi-cargo intracellular delivery
Source: Sci Rep. 2018 Aug 22;8:12538. doi: 10.1038/s41598-018-30790-2 (PMC6105642; doi:10.1038/s41598-018-30790-2)
Supplement: Supplementary file 2 — Supplementary Tables [file 41598_2018_30790_MOESM2_ESM.pdf]

Supplementary Tables

A platform for discovery of functional cell-penetrating peptides for efficient multi-cargo intracellular delivery

Katrin Hoffmann[#], Nadia Milech[#,*], Suzy M Juraja[#], Paula T Cunningham[#], Shane R Stone, Richard W Francis, Mark Anastasas, Clinton M Hall, Tatjana Heinrich, Heique M Bogdawa, Scott Winslow, Marie N Scobie, Robert E Dewhurst, Laura Florez, Ferrer Ong, Maria Kerfoot, Danie Champain, Abbie M Adams, Susan Fletcher, Helena M Viola, Livia C Hool, Theresa Connor, Brooke AC Longville, Yew-Foon Tan, Karen Kroeger, Volker Morath, Gregory A Weiss, Arne Skerra, Richard M Hopkins and Paul M Watt.

*# Shared first authorship; * Corresponding author*

Supplementary Tables....1
Supplementary Tables: Composition of Phylomer libraries.... 2
Supplementary Table 1: Archaea library (T08).... 2
Supplementary Table 2: Pathogenic bacteria library (T09).... 2
Supplementary Table 3: Synthetic viral sequences library (T12).... 3
Supplementary Tables: Proteins.... 6
Supplementary Table 4: pET28a+ constructs; recombinant biologic cargos.... 6
Supplementary Table 5: PASylated SpyC protein.... 9
Supplementary Tables: Peptide sequences.... 10
Supplementary Table 6: CPP_SpyTag peptides.... 10
Supplementary Table 7: CPP_^{D}PMI peptides.... 12
Supplementary Table 8: CPP_PAP_Cleavable-linker peptides.... 13
Supplementary Tables: Constructs..... 14
Supplementary Table 9: pACYC184 construct (15A origin; chloramphenicol resistance); EnScape "decoy" construct.... 14
Bibliography (Supplementary Tables)....15

Supplementary Tables: Composition of Phylomer libraries

Supplementary Table 1: Archaea library (T08)

Genus species	Genome size (Kb)
Aeropyrum pernix	1670
Archeaoglobus fulgidis	2178
Haloarcula marismortui	4275
Haloferax volcanii	4013
Methanocaldococcus jannaschii	1734
Pyrococcus horikoshii	1738
Sulfolobus solfataricus	2992
Thermoplasma volcanicum	1585
Halobacterium salinarum	2571

Supplementary Table 2: Pathogenic bacteria library (T09)

Genus species	Genome size (Kb)
Bordetella pertussis	*4086*
Borrelia burgdorferi	1444
Campylobacter jejuni	1641
Haemophilus influenzae	1830
Helicobacter pylori	1667
Neisseria menigitidis	2195
Porphyromonas gingivalis	2343
Pseudomonas aeruginosa	6264
Salmonella enterica	4951
Staphylococcus aureus	2903
Streptococcus pyogenes	1852
Acinetobacter baumannii	3977
Aeromonas hydrophila	4744
Bacillus cereus	5433
Clostridium difficile	4298
Clostridium perfringens	3257

Corynebacterium diphtheriae	2489
Legionella pneumophila	3398
Listeria monocytogenes	2945
Mycobacterium avium	4830
Mycobacterium tuberculosis	4420
Neisseria gonorrhoeae	2223

Supplementary Table 3: Synthetic viral sequences library (T12)

Virus	Gene	Protein function
Influenza A virus (H1N1)	Neuraminidase	Envelope, host cell attachment
	Hemagglutinin	Envelope, host cell attachment
Dengue type 1 virus	Capsid protein	Structural
	Protein M	Envelope, structural
	Protein E	Envelope, host cell attachment
	Non-structural protein 1	Virus replication and regulation of host immune response
	Non-structural protein 2A	Viral RNA replication and capsid assembly
	serine protease NS2B	Cofactor for the serine protease function of NS3
	Serine protease NS3	Cleaves the virus genome polyprotein into component proteins
	Non-structural protein 4A;	Viral replication
	Non-structural protein 4B;	Regulation of host immune response
	RNA-directed RNA polymerase NS5	Replication of viral genome
Human herpesvirus 4 (Ebstein-Barr virus)	Glycoprotein 42 (gp42)	Envelope, host cell attachment
	BMRF2	Envelope, host cell attachment
	gp350 (BLLF1)	Envelope, host cell attachment
Human herpes virus 8 (Karposis sarcoma virus)	Envelope glycoprotein B (gB)	Envelope, host cell attachment
Zaire ebola virus	GP1	Envelope, host cell attachment
	GP2	Envelope, host cell fusion
Lake Victoria marburgvirus	GP1	Envelope, host cell attachment
	GP2	Envelope, host cell fusion
Newcastle disease virus	Fusion glycoprotein F0	Envelope, host cell fusion
Measles virus	Hemagglutinin glycoprotein	Envelope, host cell attachment
Human respiratory syncytial virus B	Fusion glycoprotein F0	Envelope, host cell fusion
Vesicular stomatitis Indiana virus	Glycoprotein G	Envelope, host cell attachment
Influenza C virus	Hemagglutinin-esterase-fusion glycoprotein	Envelope, host cell fusion

Adeno-associated virus 2	VP1 (Virion protein 1)	Structural capsid protein and cell attachment
Foot-and-mouth virus	Protein Leader	Protease: cleaves virus genome polyprotein into component proteins
	Protein VP2	Structural, capsid, cell attachment
	Protein VP3	Structural, capsid, cell attachment
	Protein VP4	Structural, capsid, cell attachment
	Protein VP1	Structural, capsid, cell attachment
	Protein2AB	Effects host cell membrane permeability
	Protein 2C	Effects intracellular membranes
	Protein 3A	Virus genome replication
	Protein 3B	Virus genome replication
	Protein 3C	Virus genome replication
	Protein 3D	Virus genome replication
Hepatitis A virus	Protein Leader	Protease: cleaves genome polyprotein into component proteins
	Protein VP1	Structural, capsid, cell attachment
	Protein VP2	Structural, capsid, cell attachment
	Protein VP3	Structural, capsid, cell attachment
	Protein VP4	Structural, capsid, cell attachment
	Protein2A	Effects host cell membrane permeability
	Protein2B	Effects host cell membrane permeability
	Protein 2C	Effects intracellular membranes
	Protein 3A	Virus genome replication
	Protein 3B	Virus genome replication
	Protein 3C	Virus genome replication
	Protein 3D	Virus genome replication
Human parechovirus 1 (echovirus 22)	Protein VP0	Structural, capsid, cell attachment
	Protein VP1	Structural, capsid, cell attachment
	Protein VP2	Structural, capsid, cell attachment
	Protein VP3	Structural, capsid, cell attachment
	Protein2AB	Effects host cell membrane permeability
	Protein 2C	Effects intracellular membranes
	Protein 3A	Virus genome replication
	Protein 3B	Virus genome replication
	Protein 3C	Virus genome replication
	Protein 3D	Virus genome replication
	Protein 3D	Virus genome replication
Simian Virus 40	VP1	Structural, capsid, cell attachment and entry
	VP2	Structural, capsid, viral particle assembly

	VP3	Structural, capsid, viral particle assembly
Rotavirus A	VP4	Structural, capsid, cell attachment
	VP7	Structural, capsid, cell attachment
Reovirus type 1	Attachment protein σ1	Structural, capsid, cell attachment
Avian leukosis virus RSA (RSV-SRA) / Rous sarcoma virus	Envelope glycoprotein gp95	Envelope, viral-host cell fusion
Human immunodeficiency virus 1	Envelope glycoprotein gp160 (gp120 and gp41 chains)	Envelope, host cell attachment and viral-host cell fusion
Sindbis virus	Capsid protein	Structural, capsid, viral assembly
	E3 protein (Spike glycoprotein E3)	Envelope, function unknown
	E2 envelope glycoprotein (spike glycoprotein E2);	Envelope, host cell attachment
	6K protein;	Envelope, host cell membrane permeabilization
	E1 envelope glycoprotein (Spike glycoprotein E1)	Envelope, virus-host cell fusion
Hepatitis B virus	Large envelope protein (L glycoprotein or S protein)	Envelope, host cell attachment
Human herpes virus 1 (HSV-1)	Envelope glycoprotein C (gC)	Envelope, host cell attachment
	Envelope glycoprotein B (gB)	Envelope, host cell attachment
Human herpes virus 5 (CMV)s	Envelope glycoprotein B (gB)	Envelope, host cell attachment and entry
Human adenovirus C serotype 2 (HAdv-2)	Fiber protein	Structural, capsid, host cell attachment
Human papilloma virus 16	Major capsid protein L1	Structural, capsid, cell entry
	Minor capsid protein L2	Structural, capsid, cell entry

Supplementary Tables: Proteins

CPP_EBD_S11 and CPP_TRX_S11 recombinant proteins were constructed in pET28a+ and expressed as previously described[1].

Supplementary Table 4: pET28a+ constructs; recombinant biologic cargos

DNA sequences were synthesized and cloned (ATUM, USA) into the *Nco*I and *Xho*I sites of pET28a^{+} vector (Merck Millipore).
Protein references: SpyCatcher (GenBank: JQ478411.1), Bouganin (GenBank: AAL35962.1; expressed protein is Bouganin 27-276aa), β-lactamase (NCBI Reference Sequence: WP_015058867.1; expressed protein is BLA 24-286 aa).
Protein sequences for PAP[2], Omomyc[3,4] and Affibody$_{EGFR-1907}$[5] were sourced from the literature.

Protein expressed	Sequence with annotations	kDa	pI	Notes
SpyC_PAP	MGHHHHHHGATLEVLFQGPGGSDSATHIKFSKRDEDGKELAGATMELRDS<br>SGKTISTWISDGQVKDFYLYPGKYTFVETAAPDGYEVATAITFTVNEQGQ<br>VTVNGKATKGGSGTGATSGKLAKLAKKLAKLAK	14.0	8.8	SpyCatcher-PAP fusion protein. SpyCatcher sequence in light blue; PAP sequence in lavender; His tag used in purification in blue. Can be conjugated to SpyTag-containing peptides. Used in PAP cell viability assays.
SpyC_BLA	MGHHHHHHGATLEVLFQGPGGSGSDSATHIKFSKRDEDGKELAGATMELR<br>DSSGKTISTWISDGQVKDFYLYPGKYTFVETAAPDGYEVATAITFTVNEQ<br>GQVTVNGKATKGSHPETLVKVKDAEDQLGARVGYIELDLNSGKILESFRP<br>EERFPMMSTFKVLLCGAVLSRIDAGQEQLGRRIHYSQNDLVEYSPVTEKH<br>LTDGMTVRELCSAAITMSDNTAANLLLTTIGGPKELTAFLHNMGDHVTRL<br>DRWEPELNEAIPNDERDTTMPAAMATTLRKLLTGELLTLASRQQLIDWME<br>ADKVAGPLLRSALPAGWFIADKSGAGERGSRGIIAALGPDGKPSRIVVIY<br>TTGSQATMDERNRQIAEIGASLIKHWQLGSASGTTGATSGEF	42.4	5.6	SpyCatcher_β-lactamase fusion protein. SpyCatcher sequence in light blue; BLA sequence in red; His tag used in purification in blue. Can be conjugated to SpyTag-containing peptides. Used in β-lactamase bioassays. Gives high yield of soluble protein.
Boug	MGSSHHHHHHGGSYNTVSFNLGEAYEYPTFIQDLRNELAKGTPVCQLPVT<br>LQTIADDKRFVLVDITTTSKKTVKVAIDVTDVYVVGYQDKWDGKDRAVFL<br>DKVPTVATSKLFPGVTNRVTLTFDGSYQKLVNAAKVDRKDLELGVYKLEF<br>SIEAIHGKTINGQEIAKFFLIVIQMVSEAARFKYIETEVVDRGLYGSFKP<br>NFKVLNLENNWGDISDAIHKSSPQCTTINPALQLISPSNDPWVVNKVSQI<br>SPDMGILKFKSSKGSGATAGSAATGGATGGSTS	30.9	7.8	Bouganin protein. Bouganin sequence in carmine; His tag used in purification in blue. CT sequence includes cloning site for potential addition of modular components. Used in Bouganin cell viability assays.

EGFRAffBd_SpyC	MGHHHHHHGATLEVLFQGPGGSGSVDNKFNKEMWAAWEEIRNLPNLNGWQMTAFIASLVDDPSQSANLLAEAKKLNDAQAPKGTGSGATAGSAATGGATGGSDSATHIKFSKRDEDGKELAGATMELRDSSGKTISTWISDGQVKDFYLYPGKYTFVETAAPDGYEVATAITFTVNEQGQVTVNGKATKGGAGSWSHPQFEKG	21.4	5.7	EGFR Affibody_SpyCatcher fusion protein. EGFR Affibody (1907) sequence in purple; SpyCatcher sequence in light blue; His tag used in purification in blue. Can be conjugated to SpyTag-containing peptides. Used in Bouganin cell viability assays.
EGFRAffBd-Boug_SpyC	MGHHHHHHGATLEVLFQGPGGSGSVDNKFNKEMWAAWEEIRNLPNLNGWQMTAFIASLVDDPSQSANLLAEAKKLNDAQAPKGTGSGTGSATSGSLAGSGATAGTGSGYNTVSFNLGEAYEYPTFIQDLRNELAKGTPVCQLPVTLQTIADDKRFVLVDITTTSKKTVKVAIDVTDVYVVGYQDKWDGKDRAVFLDKVPTVATSKLFPGVTNRVTLTFDGSYQKLVNAAKVDRKDLELGVYKLEFSIEAIHGKTINGQEIAKFFLIVIQMVSEAARFKYIETEVVDRGLYGSFKPNFKVLNLENNWGDISDAIHKSSPQCTTINPALQLISPSNDPWVVNKVSQISPDMGILKFKSSKGSGATAGSAATGGATGGSDSATHIKFSKRDEDGKELAGATMELRDSSGKTISTWISDGQVKDFYLYPGKYTFVETAAPDGYEVATAITFTVNEQGQVTVNGKATKGGAGSWSHPQFEKG	51.2	6.1	EGFR Affibody_Bouganin_SpyCatcher fusion protein. EGFR Affibody (1907) sequence in purple; Bouganin sequence in carmine; SpyCatcher sequence in light blue; His tag used in purification in blue. Can be conjugated to SpyTag-containing peptides. Used in Bouganin cell viability assays.
SpyC	MASHHHHHHGATLEVLFQGPGGSDSATHIKFSKRDEDGKELAGATMELRDSSGKTISTWISDGQVKDFYLYPGKYTFVETAAPDGYEVATAITFTVNEQGQVTVNGKATKGTSGAGKPIPNPLLGLDST	17.3	9.7	SpyCatcher protein. SpyCatcher sequence in light blue; His tag used in purification in blue; V5 tag underlined. Can be conjugated to SpyTag-containing peptides. Used in mechanism uptake assays conjugated to Napthofluorscein-labelled peptides.
Omomyc	*GPGGS*GTGATSDTEENVKRRTHNVLERQRRNELKRSFFALRDQIPELENNEKAPKVVILKKATAYILSVQAETQKLISEIDLLRKQNEQLKHKLEQLRNSCA	11.6	9.6	Omomyc protein. Omomyc sequence in maroon; 5aa at the NT are residual sequence from cleavage of Thioredoxin (Trx) solubility tag and are italicized. Used in Omomyc cell viability assays.
1746c27_Omomyc	*GPGGS*KKKKQPPKPKKPKTQEKKKKQPPKPKRHHHHHHGSGTGATSGSDTEENVKRRTHNVLERQRRNELKRSFFALRDQIPELENNEKAPKVVILKKATAYILSVQAETQKLISEIDLLRKQNEQLKHKLEQLRNSCATSGAGKPIPNPLLGLDST	17.7	10.3	1746c27_Omomyc fusion protein. CPP sequence in orange; Omomyc sequence in maroon; His tag used for purification in blue; V5 tag underlined; 5aa at the NT are residual sequence from cleavage of

				Thioredoxin (Trx) solubility tag and are italicized. Used in Omomyc cell viability assays.

NT = Amino terminus; CT = Carboxy terminus.

Supplementary Table 5: PASylated SpyC protein

PAS_SpyC was expressed from vector pASK37 and the construct was cloned in two parts. First, the DNA sequence of the SpyCatcher (SpyC) section coding sequence was synthesized with an N' linker (MPACSSA) that contains a *Sap*I recognition site for the insertion of P/A gene sequences[6] and a cysteine residue for later maleimide-conjugation, followed by a minimized SpyC[7] (comprising Asp48 to Gly130[8]) which was synthesized with a C-terminal *Strep*-tag II[9] followed by a *Hin*dIII recognition site. The synthesized fragment was cloned on the vector pASK37 using *Nde*I and *Hin*dIII restriction sites to form an interim SpyC construct. Second, the interim SpyC construct was linearized using *Sap*I and a P/A#1(600) gene cassette was inserted into the N-terminal linker upstream of the SpyC gene.

Protein expressed	Sequence with annotations	kDa	pI	Notes
PAS_SpyC	MPACSSAAPAAPAPAAPAAPAPAAPAAAPAAPAPAAPAAP<br>APAAPAAAPAAPAPAAPAAPAPAAPAAAPAAPAPAAPAAP<br>APAAPAAAPAAPAPAAPAAPAPAAPAAAPAAPAPAAPAAP<br>APAAPAAAPAAPAPAAPAAPAPAAPAAAPAAPAPAAPAAP<br>APAAPAAAPAAPAPAAPAAPAPAAPAAAPAAPAPAAPAAP<br>APAAPAAAPAAPAPAAPAAPAPAAPAAAPAAPAPAAPAAP<br>APAAPAAAPAAPAPAAPAAPAPAAPAAAPAAPAPAAPAAP<br>APAAPAAAPAAPAPAAPAAPAPAAPAAAPAAPAPAAPAAP<br>APAAPAAAPAAPAPAAPAAPAPAAPAAAPAAPAPAAPAAP<br>APAAPAAAPAAPAPAAPAAPAPAAPAAAPAAPAPAAPAAP<br>APAAPAAAPAAPAPAAPAAPAPAAPAAAPAAPAPAAPAAP<br>APAAPAAAPAAPAPAAPAAPAPAAPAAAPAAPAPAAPAAP<br>APAAPAAAPAAPAPAAPAAPAPAAPAAAPAAPAPAAPAAP<br>APAAPAAAPAAPAPAAPAAPAPAAPAAAPAAPAPAAPAAP<br>APAAPAAAPAAPAPAAPAAPAPAAPAAAPAAPAPAAPAAP<br>APAAPAADSATHIKFSKRDEDGKELAGATMELRDSSGKTI<br>STWISDGQVKDFYLYPGKYTFVETAAPDGYEVATAITFTV<br>NEQGQVTVNGSAWSHPQFEK	59.0	4.8	PAS_SpyC comprises an N-terminal Cys-residue that was incorporated to enable the conjugation of small molecule targeting ligands (as a negative control, the Cys residue was conjugated to aminoethyl-maleimide). The PAS(600) moiety comprising 600 P/A (Pro and Ala) residues served for plasma half-life extension according to the PASylation technology. The SpyCatcher was employed in its minimal version and the *Strep*-tag II was incorporated for detection and/or purification purposes. The resulting multi-functional fusion protein can be easily conjugated to SpyTag-containing peptides.

Supplementary Tables: Peptide sequences

Supplementary Table 6: CPP_SpyTag peptides

Peptide Name	Sequence	Notes
TAT_SpyT	GRKKRRQRRR**GAS**<u>AHIVMVDAYKPTKG</u>	TAT sequence. NT Acetylated, CT amidated; GAS linker in bold; canonical CPP sequence in blue; SpyTag underlined.
Penetratin_SpyT	RQIKIWFQNRRMKWKK**GAS**<u>AHIVMVDAYKPTKG</u>	Penetratin sequence. NT Acetylated, CT amidated; GAS linker in bold; canonical CPP sequence in blue; SpyTag underlined.
1746_SpyT	PLKPKKPKTQEKKKKQPPKPKKPKTQEKKKKQPPKPKR**GAS**<u>AHIVMVDAYKPTKG</u>	Parental 1746 sequence. NT Acetylated, CT amidated; GAS linker in bold; CPP sequence in orange; SpyTag underlined.
1746del_SpyT	KTQEKKKKQPPKPKKPKTQEKKKKQPPKPKR**GAS**<u>AHIVMVDAYKPTK</u>	NT truncation, NT -7aa. NT Acetylated, CT amidated; GAS linker in bold; CPP sequence in orange; SpyTag underlined.
1746c27_SpyT	KKKKQPPKPKKPKTQEKKKKQPPKPKR**GAS**<u>AHIVMVDAYKPTKG</u>	NT truncation, NT -11aa. NT Acetylated, CT amidated; GAS linker in bold; CPP sequence in orange; SpyTag underlined.
1746-SAR1_SpyT	PKKPKTQEKKKKQPPK**GAS**<u>AHIVMVDAYKPTKG</u>	NT & CT truncation, NT -3aa, CT -19aa. NT Acetylated, CT amidated; GAS linker in bold; CPP sequence in orange; SpyTag underlined
1746-SAR2_SpyT	PLKPKKPKTQEKKKKQPPKPK**GAS**<u>AHIVMVDAYKPTKG</u>	CT truncation, CT -17aa. NT Acetylated, CT amidated; GAS linker in bold; CPP sequence in orange; SpyTag underlined.
1746-SAR3_SpyT	PLKPKKPKTQEKKKKQPPKPKKPKT**GAS**<u>AHIVMVDAYKPTKG</u>	CT truncation, CT -13aa. NT Acetylated, CT amidated; GAS linker in bold; CPP sequence in orange; SpyTag underlined.
1746-SAR4_SpyT	PKTQEKKKKQPPKPKKPKTQEKKKKQP**GAS**<u>AHIVMVDAYKPTKG</u>	NT & CT truncation, NT -6aa, CT -5aa. NT Acetylated, CT amidated; GAS linker in bold; CPP sequence in orange; SpyTag underlined.
1746-SAR5_SpyT	KKQPPKPKKPKTQEKKKKQPPKPKR**GAS**<u>AHIVMVDAYKPTKG</u>	NT truncation, NT -13aa. NT Acetylated, CT amidated; GAS linker in bold; CPP sequence in orange; SpyTag underlined.
1746-SAR6_SpyT	PRQKKQAPKQPPKPKKPKTQEKKKKQP**GAS**<u>AHIVMVDAYKPTKG</u>	NT extension, NT +10aa; CT truncation, CT -21aa. NT Acetylated, CT amidated; GAS linker in bold; CPP sequence in orange; SpyTag underlined.

1746-SAR7_SpyT	TKTQEKKKKQTTKTKKTKTQEKKKKQT**GAS**AHIVMVDAYKPTKG	NT & CT truncation, NT -6aa, CT -5aa; mutation, P to T. NT Acetylated, CT amidated; GAS linker in bold; CPP sequence in orange; SpyTag underlined.
1746-SAR9_SpyT	PKTQEAAAAQPPKPKKPKTQEAAAAQP**GAS**AHIVMVDAYKPTKG	NT & CT truncation, NT -6aa, CT -5aa; mutation, KKKK to AAAA. NT Acetylated, CT amidated; GAS linker in bold; CPP sequence in orange; SpyTag underlined.
1746-SAR12_SpyT	PLKPKKPKTQEKKKKQPPKPKKPKTQEKKKKQPPKPK**GAS**AHIVMVDAYKPTKG	CT truncation, CT -1aa. NT Acetylated, CT amidated; GAS linker in bold; CPP sequence in orange; SpyTag underlined.
1746-SAR13_SpyT	KPKKPKTQEKKKKQPPKPKKPKTQEKKKKQPPKPKR**GAS**AHIVMVDAYKPTKG	NT truncation, NT -2aa. NT Acetylated, CT amidated; GAS linker in bold; CPP sequence in orange; SpyTag underlined.
1746-SAR14_SpyT	KKPKTQEKKKKQPPKPKKPKTQEKKKKQPPKPKR**GAS**AHIVMVDAYKPTKG	NT truncation, NT -4aa. NT Acetylated, CT amidated; GAS linker in bold; CPP sequence in orange; SpyTag underlined.
1746-SAR15_SpyT	KKQPPKPKKPKTQEKKKKQPPKPKKPKTQEKK**GAS**AHIVMVDAYKPTKG	NT extension, NT +3aa; CT truncation, CT -19aa. NT Acetylated, CT amidated; GAS linker in bold; CPP sequence in orange; SpyTag underlined.
1746-SAR16_SpyT	TLKTKKTKTQEKKKKQTTKTKKTKTQEKKKKQTTKTKR**GAS**AHIVMVDAYKPTKG	Full length; mutation, P to K. NT Acetylated, CT amidated; GAS linker in bold; CPP sequence in orange; SpyTag underlined.
1746-SAR17_SpyT	PLRPRRPRTQERRRRQPPRPRRPRTQERRRRQPPRPRR**GAS**AHIVMVDAYKPTKG	Full length; mutation, K to R. NT Acetylated, CT amidated; GAS linker in bold; CPP sequence in orange; SpyTag underlined.

NT = Amino terminus; CT = Carboxy terminus.

Supplementary Table 7: CPP_^{D}PMI peptides

Peptide Name	Sequence	Notes
^{D}PMIα	tnwyanlekllr	^{D}PMIα sequence. NT Acetylated, CT amidated; GAS linker in bold; ^{D}PMI underlined with D-form amino acids in lowercase.
TAT_^{D}PMIα	GRKKRRQRRR**GAS**tnwyanlekllr	TAT_^{D}PMIα sequence. NT Acetylated, CT amidated; GAS linker in bold; canonical CPP sequence in blue; ^{D}PMI underlined with D-form amino acids in lowercase.
1746_^{D}PMIα	PLKPKKPKTQEKKKKQPPKPKKPKTQEKKKKQPPKPKR**GAS**tnwyanlekllr	1746_^{D}PMIα sequence. NT Acetylated, CT amidated; GAS linker in bold; CPP sequence in orange; ^{D}PMI underlined with D-form amino acids in lowercase.

NT = Amino terminus; CT = Carboxy terminus.

Supplementary Table 8: CPP_PAP_Cleavable-linker peptides

Peptide Name	Sequence	Notes
1746c27_PAP_Ba_V5_SpyT	KKKKQPPKPKKPKTQEKKKKQPPKPKR**GAS**KLAKLAKKLAKLAKC(Mcpa)GAV(Cit)AGGKPIPNPLLGLDST**GAS**AHIVMVDAYKPTKG	**Cathepsin-B: Valine-Citrulline Linker**[10]. NT Acetylated, CT amidated; GAS linkers in bold; CPP sequence in orange; SpyTag underlined; PAP sequence in lavender; V5 tag in blue except for the first amino acid (G) which is in red and underlined as it overlaps with the protease cleavage site. Full Cathepsin-B protease cleavage site is indicated in red and underlined. Cit indicates Citrulline non-natural amino acid. Mcpa stands for mercaptopropionic acid.
1746c27_PAP_BF_V5_SpyT	KKKKQPPKPKKPKTQEKKKKQPPKPKR**GAS**KLAKLAKKLAKLAKC(Mcpa)GFKFLGGKPIPNPLLGLDST**GAS**AHIVMVDAYKPTKG	**Cathepsin-B: FKFL Linker**[11]. NT Acetylated, CT amidated; GAS linkers in bold; CPP sequence in orange; SpyTag underlined; PAP sequence in lavender; V5 tag in blue except for the first amino acid (G) which is in red and underlined as it overlaps with the protease cleavage site. Full Cathepsin-B protease cleavage site is indicated in bold and underlined. Mcpa stands for mercaptopropionic acid.
1746c27_PAP_Furin_V5_SpyT	KKKKQPPKPKKPKTQEKKKKQPPKPKR**GAS**KLAKLAKKLAKLAK*C*(Mcpa)RKKRSVGKPIPNPLLGLDST**GAS**AHIVMVDAYKPTKG	**Furin Linker**[12,13]. (MEROPS site: https://www.ebi.ac.uk/merops/cgi-bin/pepsum?id=S08.071;type=P) NT Acetylated, CT amidated; GAS linkers in bold; CPP sequence in orange; SpyTag underlined; PAP sequence in lavender; V5 tag in blue except for the first amino acid (G) which is in red and underlined as it overlaps with the protease cleavage site. Full Furin protease cleavage site is indicated in bold and underlined. Mcpa stands for mercaptopropionic acid.

NT = Amino terminus; CT = Carboxy terminus.

Supplementary Tables: Constructs.

Supplementary Table 9: pACYC184 construct (15A origin; chloramphenicol resistance); EnScape "decoy" construct

Protein reference: SUMO (NCBI Reference Sequence: NP_010798.1; expressed protein is SMT3 1-98 aa)

Protein expressed	Sequence with annotations	kDa	pI	Notes
SUMO_Avi3	MSDSEVNQEAKPEVKPEVKPETHINLKVSDGSSEIFFKIK KTTPLRRLMEAFAKRQGKEMDSLRFLYDGIRIQADQTPED LDMEDNDIIEAHREQIGGSSGLNDIFEAQKIEWHEGGSGL NDIFEAQKIEWHEGGSGLNDIFEAQKIEWHE	17.2	4.7	97 aa SUMO cds followed by 3 consecutive Avitags, each "domain" separated by GSS linkers.<br>Co-transformed into phage host cell strain BLT5615 to produce non-biotinylated T7_avi phage particles. The leaky tac promoter produces a SUMO protein with 3 consecutive avitag sequences. This "decoy" protein is strongly biotinylated thus depleting the *E. coli* biotinylation machinery before T7 phage production is induced by the addition of IPTG.

Bibliography (Supplementary Tables)

1. Milech, N. *et al.* GFP-complementation assay to detect functional CPP and protein delivery into living cells. *Sci Rep* **5,** 18329 (2015).
2. Ellerby, H. M. *et al.* Anti-cancer activity of targeted pro-apoptotic peptides. *Nat Med* **5,** 1032–1038 (1999).
3. Soucek, L. *et al.* Design and properties of a Myc derivative that efficiently homodimerizes. *Oncogene* **17,** 2463–2472 (1998).
4. Soucek, L. *et al.* Omomyc, a potential Myc dominant negative, enhances Myc-induced apoptosis. *Cancer Res* **62,** 3507–3510 (2002).
5. Friedman, M. *et al.* Directed evolution to low nanomolar affinity of a tumor-targeting epidermal growth factor receptor-binding affibody molecule. *J Mol Biol* **376,** 1388–1402 (2008).
6. Binder, U. & Skerra, A. PASylation®: A versatile technology to extend drug delivery. *Curr Opin Colloid Interface Sci* **31,** 10–17 (2017).
7. Zakeri, B. *et al.* Peptide tag forming a rapid covalent bond to a protein, through engineering a bacterial adhesin. *P Natl Acad Sci USA* **109,** E690–7 (2012).
8. Li, L., Fierer, J. O., Rapoport, T. A. & Howarth, M. Structural analysis and optimization of the covalent association between SpyCatcher and a peptide Tag. *J Mol Biol* **426,** 309–317 (2014).
9. Schmidt, T. G., Koepke, J., Frank, R. & Skerra, A. Molecular interaction between the Strep-tag affinity peptide and its cognate target, streptavidin. *J Mol Biol* **255,** 753–766 (1996).
10. Liang, L. *et al.* Novel cathepsin B-sensitive paclitaxel conjugate: Higher water solubility, better efficacy and lower toxicity. *J Control Release* **160,** 618–629 (2012).
11. Chu, D. S. H., Johnson, R. N. & Pun, S. H. Cathepsin B-sensitive polymers for compartment-specific degradation and nucleic acid release. *J Control Release* **157,** 445–454 (2012).
12. Thomas, G. Furin at the cutting edge: from protein traffic to embryogenesis and disease. *Nat Rev Mol Cell Biol* **3,** 753–766 (2002).
13. Rawlings, N. D., Barrett, A. J. & Finn, R. Twenty years of the MEROPS database of proteolytic enzymes, their substrates and inhibitors. *Nucleic Acids Res* **44,** D343–50 (2016).
